# Development of Novel Silyl Cyanocinnamic Acid Derivatives as Metabolic Plasticity Inhibitors for Cancer Treatment

**DOI:** 10.1038/s41598-019-54709-7

**Published:** 2019-12-04

**Authors:** Grady L. Nelson, Conor T. Ronayne, Lucas N. Solano, Sravan K. Jonnalagadda, Shirisha Jonnalagadda, Jon Rumbley, Jon Holy, Teresa Rose-Hellekant, Lester R. Drewes, Venkatram R. Mereddy

**Affiliations:** 1 Integrated Biosciences Graduate Program, University of Minnesota, Duluth, MN 55812 USA; 2 Department of Chemistry and Biochemistry, University of Minnesota Duluth, Duluth, MN 55812 USA; 3 Department of Biomedical Sciences, Medical School Duluth, University of Minnesota, Duluth, MN 55812 USA; 4 Department of Pharmacy Practice & Pharmaceutical Sciences, University of Minnesota, Duluth, MN 55812 USA

**Keywords:** Drug discovery, Oncology

## Abstract

Novel silyl cyanocinnamic acid derivatives have been synthesized and evaluated as potential anticancer agents. *In vitro* studies reveal that lead derivatives **2a** and **2b** have enhanced cancer cell proliferation inhibition properties when compared to the parent monocarboxylate transporter (MCT) inhibitor cyano-hydroxycinnamic acid (CHC). Further, candidate compounds exhibit several-fold more potent MCT1 inhibition properties as determined by lactate-uptake studies, and these studies are supported by MCT homology modeling and computational inhibitor-docking studies. *In vitro* effects on glycolysis and mitochondrial metabolism also illustrate that the lead derivatives **2a** and **2b** lead to significant effects on both metabolic pathways. *In vivo* systemic toxicity and efficacy studies in colorectal cancer cell WiDr tumor xenograft demonstrate that candidate compounds are well tolerated and exhibit good single agent anticancer efficacy properties.

## Introduction

Tumor growth requires increased energetic and biosynthetic demands in a microenvironment that typically varies in oxygen and nutrient distribution due to differences in tumor cell proximity to blood vessels^1,2^. A common strategy employed by malignant cells to deal with these conditions involves reprogramming their metabolism toward aerobic glycolysis (Warburg Effect)^3–6^. This molecular reprogramming is a critical hallmark of solid tumors and is an important target for cancer treatment^1–6^.

Increased glycolysis results in the generation of byproducts such as lactic acid leading to an initial decrease in the internal pH; compensated by the elevated expression of monocarboxylate transporters (MCTs) and other transporters that efflux the acidic components and decrease the external pH^7,8^. Interestingly, it has been recently shown that extracellular lactate can be taken up by MCTs in neighboring proliferating cancer cells (Reverse Warburg Effect) and utilized in the mitochondrial TCA cycle and oxidative phosphorylation (OxPhos)^9–12^. Exchanges between anabolic and catabolic compartments facilitate tumor growth and have also been shown to promote resistance to chemo- and radiation therapies^13^. These metabolic specializations are distinct from normal cells and the resulting upregulation of numerous glycolytic enzymes and transporters provide an opportunity for pharmacological intervention^12,14^. Similar to glycolysis, mitochondrial OxPhos also plays an integral part in ATP production and contributes to the biosynthetic demands of rapidly proliferating cancer cells. Depending on nutrient availability, tumor cells can develop metabolic plasticity switching between glycolysis and OxPhos or exhibit an intermediate phenotype to maximize their chances of survival^12,14^.

Metabolic plasticity is responsible for maintaining energetic homeostasis and to facilitate this plasticity, cancer cells increase the expression of MCTs. MCTs regulate the transport of lactate, pyruvate as well as other ketone bodies to support the metabolic demands in cancer cells^15,16^. Specifically, MCTs 1 and 4 are the primary transporters involved in transporting glycolytic by-products in and out of the cells, respectively; supplying oxidative cells with TCA metabolites, and circumventing a rapid decrease in intracellular pH^12,16,17^. MCT1 and 4 overexpression is a hallmark of cancer progression and provides a promising target for chemotherapeutics and recent studies have shown that targeting these transporters effectively reduces the rate of tumor progression *in vivo*^18–25^.

The small molecule α-cyano-4-hydroxycinnamic acid (CHC, **1**) has been traditionally used as an MCT inhibitor for studying cellular and biochemical functions^26^. CHC and related α-cyanocinnamic acids have also been shown to inhibit the mitochondrial pyruvate carrier, an important molecular shuttle of pyruvate in the inner mitochondrial membrane to support TCA cycle and OxPhos^27^. However, the therapeutic potential of CHC is hindered by its lack of efficacy at low concentrations and high dose requirement for significant anticancer efficacy *in vivo*. Therefore, increasing the efficacy of CHC-based compounds to simultaneously target TCA cycle and OxPhos constitute novel chemotherapeutic strategy.

In this regard, we have replaced the 4-hydroxy group in CHC with N,N-dialkyl/aryl groups that has resulted in low nanomolar potency towards lactate uptake in both MCT1 and MCT4 expressing cell lines^18,20^. Similarly, N,N-dialkyl carboxy coumarins were also found by us^19^ and others^21^ to be highly potent toward lactate uptake inhibition in MCT1 expressing cells. Although our first generation N,N-dialkyl/aryl CHC compounds are highly potent inhibitors, but *in vivo* efficacy studies required high doses (~50 mg/kg) for significant tumor growth inhibition in MCT1 expressing WiDr and MCT4 expressing MDA-MB-231 tumor models. *In vivo* pharmacokinetic analysis indicated that these compounds are rapidly eliminated with biological half-lives of <1 hr^18^. We attribute this to unsubstituted N,N-diphenyl groups and N,N-dialkyl groups which are metabolically vulnerable to CYP450 enzymatic action and subsequent elimination.

Silyl structural units such as *tert-*butyldiphenylsilyl (TBDPS) and *tert-*butyldimethylsilyl (TBS) ethers have long been used as hydroxyl protecting groups in organic chemistry due to the flexibility and stability under different pH conditions^28,29^. Recently, silyl ethers have been investigated as pharmacological tools due to their lipophilicity and high metabolic stability when administered *in vivo*^30^. Diverse functionality on the silicon atom results in varying metabolic and chemical stability, and the sterically hindered TBDPS group exhibits the highest stability^28,29^. Hence, we envisioned that introduction of an acid stable TBDPS ether on CHC phenolic hydroxyl group would increase its lipophilicity, metabolic stability, and ability to influence mitochondrial function while also retaining MCT inhibitory characteristics. In this regard, we synthesized and evaluated novel silyl-CHC compounds for their *in vitro* MCT1 inhibitory properties, *in vitro* effects on cancer cell proliferation and metabolism, and *in vivo* safety and efficacy in a WiDr tumor xenograft model. The lead candidate compounds exhibited enhanced MCT1 and cancer cell proliferation inhibition properties, led to glycolysis and mitochondrial dysfunction, and showed significant *in vivo* tumor growth inhibitions.

## Results

### Synthesis of silylated CHCs **2a** and **2b** and un-silylated CHCs **2c** and **2d**

**Figure 1:**
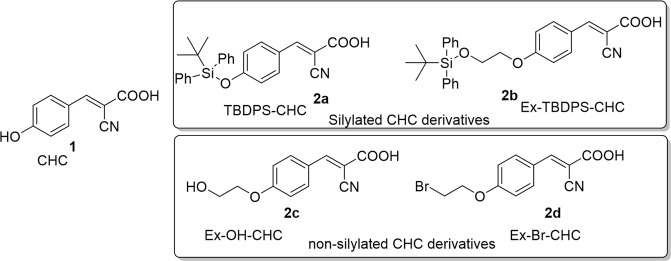
Structures of CHC **1**, silylated and non-silylated CHC derivatives **2a**–**d**

To understand the biological effects of silyl substitution on the CHC template, two representative derivatives **2a** and **2b** were synthesized (Supp. Info Fig. S1). The derivative **2a** is a silyl group containing TBDPS attached directly to CHC (TBDPS-CHC, Fig. 1). The derivative **2b** is also a silyl group containing TBDPS, which contains a 2-carbon spacer ethyl group (Ex-TBDPS-CHC, Fig. 1). The compounds **2c** (Ex-OH-CHC) and **2d** (Ex-Br-CHC) were synthesized as non-silylated analogs of extended derivative **2b** to demonstrate the importance of the silyl groups in providing biological activity (Fig. 1). The derivative **2c** contains polar hydroxy substitution whereas **2d** is a non-polar halogenated homolog of **2b**. The biological effects of parent compound CHC **1** and the four synthetic derivatives **2a–d** were then evaluated.

### *In vitro* cell proliferation inhibition studies of **2a**–**2d**

**Figure 2:**
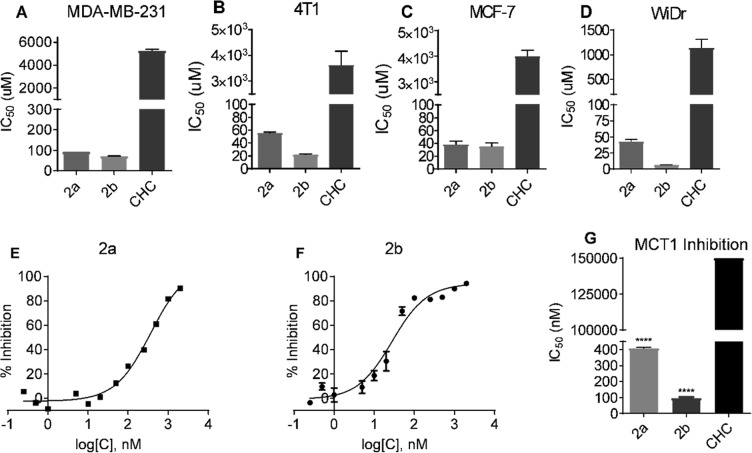
MTT cell proliferation and MCT1 inhibition IC_50_ values of candidate compounds: (**A**–**D**) represent MTT IC_50_ values in (**A**) MCF7, (**B**) 4T1, (**C**) WiDr, and (**D**) MDA-MB-231 cell lines. (**E**–**G**) represent L-[^14^C]-lactate uptake study of (**E**) compound **2a** and (**F**) compound **2b**. (**G**) MCT1 IC_50_ of compounds **2a** and **2b** in MCT1 expressing RBE4 cell line. The average ± sem of minimum three independent experimental values were calculated. Repeated measures one-way ANOVA was used to calculate statistical significance (****P < 0.0001) between test compounds and CHC.

Cell proliferation inhibition properties of candidate compounds **2a–2d** were evaluated using MTT assays on multiple cell lines. Compounds **2a** and **2b** showed highly improved cell proliferation inhibition properties with IC_50_ values of 6–93 µM compared to CHC’s IC_50_ values of 1100–5300 µM in all the cell lines tested (Table 1 and Fig. 2A–D). The non-silicon CHC derivatives **2c** and **2d** did not show significant cell proliferation inhibition at concentrations up to 500 µM. Due to solubility limitations above this concentration (0.1% DMSO in growth media), the IC_50_ values of **2c** and **2d** were not determined.

**Table 1:** MTT IC_50_ (μM) values of CHC derivatives **2a–d** in MCF7, 4T1, WiDr, and MDA-MB-231 cell lines.

Compound	MDA-MB-231	4T1	MCF7	WiDr
CHC **1**	5300 ± 130	3600 ± 300	4000 ± 130	1100 ± 96
TBDPS-CHC **2a**	93 ± 0	56 ± 1	39 ± 3	41 ± 2
Ex-TBDPS-CHC **2b**	71 ± 1	22 ± 1	35 ± 3	6 ± 1
Ex-OH-CHC **2c**	>500	>500	>500	>500
Ex-Br-CHC **2d**	>500	>500	>500	>500

*Average ± SEM of three separate experiments.

### *In vitro* MCT1 Inhibition Assay with **2a–2d**

The silylated candidate compounds **2a** and **2b** were next evaluated for *in vitro* MCT1 transport inhibition properties using an L-[^14^C]-lactate study on the MCT1 expressing RBE4 cell line as reported previously^18–20^. Both compounds **2a** and **2b** showed potent MCT1 inhibition with IC_50_ values 408 and 97 nM, respectively (Table 2, Fig. 2E–G). The parent CHC **1** exhibits weaker MCT1 inhibition properties with IC_50_ values > 150000 nM concentration. Non-silylated candidates **2c** and **2d** did not exhibit MCT1 inhibition properties at the concentrations tested (Table 2).

**Table 2:** MCT1 IC_50_ (nM) values of CHC derivatives **2a**–**d**.

Compound	MCT1
CHC **1**	>150000
TBDPS-CHC **2a**	408 ± 5
Ex-TBDPS-CHC **2b**	97 ± 7
Ex-OH-CHC **2c**	>5000
Ex-Br-CHC **2d**	>5000

*Average ± SEM of three separate experiments.

### Homology modeling of and computational inhibitor docking to human MCT1

**Figure 3:**
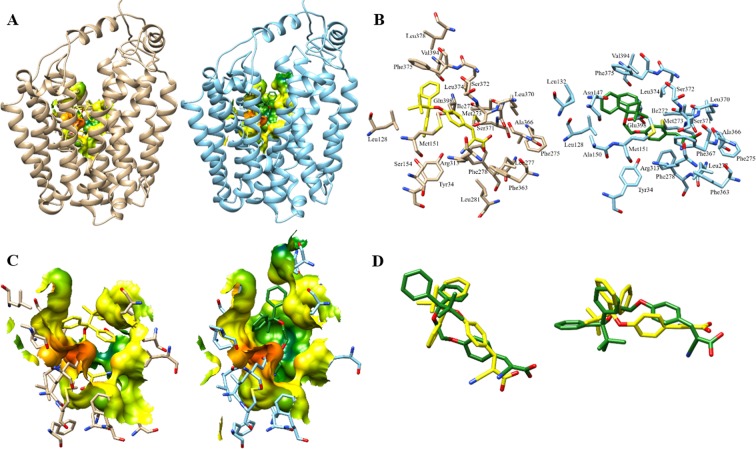
Top docking pose of candidate compounds **2a** and **2b** to homology modelled human MCT1. (**A**) Relative binding pocket of compounds **2a** (yellow) and **2b** (green) docked to human MCT1, tan and light blue respectively. Electrostatic binding surface within 4.5 Å of the inhibitors is shown. (**B**) All MCT1 residues within 4.5 Å of docked compounds **2a** (yellow) and **2b** (green). (**C**) Electrostatic surface interactions of **2a** (yellow) and **2b** (green) with human MCT, orange to red represent increasing partial negative charge while green to blue represent increasing partial positive charge and yellow is a neutral protein surface. (**D**) Overlay of top binding poses of compound **2a** (yellow) and **2b** (green) in two orientations.

To understand the potential molecular interactions of candidate inhibitors **2a** and **2b** with MCT1, homology modeling and computational docking studies were performed (Fig. 3). Five independent models of inward-open MCT1 were generated based on the human inward-open GLUT1 structure, PDB: 5eqi^31^. Due to the low sequence homology between human MCT1 and human GLUT1 (29%), consensus alignments were generated with the inclusion of additional major facilitator superfamily members and transmembrane regions were predicted by consensus topology prediction. This led to a final adjusted template and target sequence alignment for model generation. Although all models were minimized by the default optimization and molecular dynamics refinement, significant side chain rotamer variation was observed. One model was selected for further analysis based on an evaluation of the protein energy score within MODELLER and charged residue rotamer orientation in the transmembrane spans, avoiding exposure of charged residues to the putative lipid bilayer^32^. The resulting human MCT1 structure was compared to a previously reported rat MCT1 homology model also generated in an inward-open conformation but based on an *E. coli* glycerol-3-phosphate transporter (GlpT) template^33^. Overall, the registration of the transmembrane domains was remarkably similar with some residues showing a quarter to a half helical turn positioning difference and, of course, side chain rotamer differences were observed. We analyzed the residues involved in inhibitor binding between our inward-open human MCT1 structure and candidate inhibitors **2a** and **2b** (Fig. 3). These inhibitors showed much more robust inhibition of MCT1 *in vitro*, higher cell proliferation inhibition and increased ability to reduce tumor burden compared to the parent CHC or non-silylated-compounds.

Inhibitor docking to the inward-open human MCT1 homology model was carried out in Autodock Vina^34^. In order to achieve an unbiased ligand/inhibitor binding pocket search our inspection area included the entire transmembrane spanning region and extended beyond the inward-open aqueous surface of the protein. The silicon atom in **2a** and **2b** was reverted to carbon *in silico* to enable docking, resulting in a bond length change of −0.32 Å from Si-C to C-C. The structural geometries experienced no change and the overall volume change was insignificant within the respective binding poses. The best ranked docking poses of inhibitors **2a** and **2b** to MCT1 were determined to be structurally similar (Fig. 3A–D). Both compounds were found to be surrounded by several aliphatic and aromatic side chains. Contact surfaces revealed subtle differences but some of the missing surfaces in **2a** bound MCT1 are attributed to the chosen 4.5 Å cutoff and are restored at 4.6 Å+ (Fig. 3C). All amino acids contacting (<4.5 Å away, including hydrogens) either **2a** or **2b** and their positioning in the putative MCT1 binding site can be seen in Fig. 3B. Some unique contacts appearing in either **2a** or **2b** are likely due to rotamer differences between the two structures, but the extended compound (Ex-TBDPS-CHC, **2b**) does uniquely reach residues Leu132, Asn147 and Ala150 in the extended binding pocket. Within the collection of poses of the two candidate compounds occupying this site there are better matched **2a** and **2b** overlays but at the expense of significant estimated binding free energy loss (Fig. 3D). The binding affinity of the top binding pose of **2a** was estimated to be −9.2 kcal/mol for inward-open MCT1 and −9.8 kcal/mol for **2b**. In contrast, the estimated binding affinity of parent compound CHC for MCT1 was −6.4 kcal/mol for the same binding site occupancy. This difference in estimated binding affinities of CHC and candidate compounds to MCT1 equates to an approximately 160-fold higher affinity of the candidate compounds for the inward-open MCT1 homology model, consistent with experimental observation. Further, of the top 20 binding poses determined for **2a** binding to MCT1, 11 of 20 occupied the same structural binding site with minimal RMSD changes (<2 Å) while 5 of 20 poses of **2b** occupied the same MCT1 site. Conversely, only 2 of 20 poses for parent compound CHC binding to MCT1 were structurally similar to the optimal candidate compound binding site. This observation can be thought of as a surrogate for binding specificity, again consistent with experimentally determined binding affinities.

### ATP production by glycolysis and mitochondrial respiration pathways

**Figure 4:**
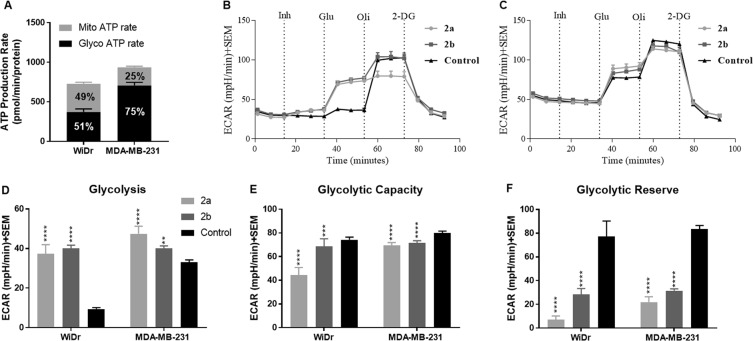
Glycolysis stress test of compounds **2a** and **2b** at 30 µM. (**A**) ATP rate assay of MDA-MB-231 and WiDr with respect to mitochondrial and glycolytic preference. Note higher percentage of mitochondrial respiration in WiDr when compared to MDA-MB-231 cell line. (**B,C**) represent glycolysis stress test profile of compounds **2a** and **2b** in (**B**) MCT1 expressing WiDr cell line, and (**C**) MCT4 expressing MDA-MB-231 cell line. Untreated cells were sequentially exposed first to test compound **2a** or **2b** (Inh) followed by glucose (Glu), oligomycin (Oli), and 2-deoxyglucose (2-DG). (**D**–**F**) represent the ECAR values of (**D**) glycolysis, (**E**) glycolytic capacity, and (**F**) glycolytic reserve in WiDr and MDA-MB-231 cell lines. The average + SEM values of three or more individual experiments were calculated. Repeated measures one-way ANOVA was used to calculate statistical significance (**P < 0.01, ****P < 0.0001) between test compounds and DMSO control (n = 3).

We then carried out the effects of candidate compounds **2a** and **2b** on glycolysis and mitochondrial respiration pathways. To first characterize relative rates of ATP production from glycolysis and mitochondrial OxPhos in each cell line, an ATP rate assay was performed. Understanding basal levels of ATP production from each respective pathway is important in interpreting differential compound effects between WiDr and MDA-MB-231 cell lines. Here, it was observed that the MCT4 expressing MDA-MB-231 cell line was highly glycolytic in nature (75% glycolysis: 25% OxPhos) and the MCT1 expressing WiDr cell line equally shared ATP production between both pathways (51% glycolysis: 49% OxPhos) (Fig. 4A).

### Evaluation of metabolic pathway profile of **2a** and **2b** using Seahorse XFe96^®^ assays

Encouraged by significant cell proliferation inhibition and potent MCT1 inhibition properties of TBDPS-CHC **2a** and Ex-TBDPS-CHC **2b**, we evaluated these candidate compounds for their effect on metabolic profiles *in vitro* using a Seahorse XFe96^®^ analyzer. Due to potential disruption in the flux of metabolically important lactate, both candidates were subjected to widely employed *Seahorse XFe96*^*®*^ based glycolysis and mitochondrial stress tests in MCT1 expressing WiDr and MCT4 expressing MDA-MB-231 cell lines.

### Glycolysis stress test

Candidate compounds **2a** and **2b** were evaluated for their effect on glycolysis, glycolytic capacity and glycolytic reserve. This assay measures extracellular acidification rate (ECAR) which is directly correlated with the rate of glycolysis. The ECAR for both cell lines in the presence of the test compounds can be seen above (Fig. 4B,C). In this assay, cells were initially starved of glucose to reduce their ECAR. Upon addition of glucose, a rise in ECAR was observed proportional to the rate of glycolysis in each cell line. An increase in basal levels of glycolysis in cells treated with test compounds compared to the controls represents a shift toward dependency on glycolysis for ATP production. Treatment with test compounds **2a** and **2b** in both WiDr and MDA-MB-231 cells significantly increased the basal glycolysis, with a more substantial increase in WiDr (Fig. 4D). It also was observed that WiDr control cultures exhibited a nearly three-fold decreased basal glycolysis when compared to MDA-MB-231 (Fig. 4D). Glycolytic capacity is the maximum rate of glycolysis the cell can attain in the absence of OxPhos as an ATP source. Both compounds **2a** and **2b** significantly reduced the glycolytic capacity in WiDr and MDA-MB-231 (Fig. 4E). The glycolytic reserve is the ability of cells to increase glycolysis when OxPhos ATP production is inhibited by oligomycin A. Here, we observed a significant decrease in the glycolytic reserve in both cell lines when treated with compound **2a** and **2b** (Fig. 4F).

### Mitochondrial stress test

**Figure 5:**
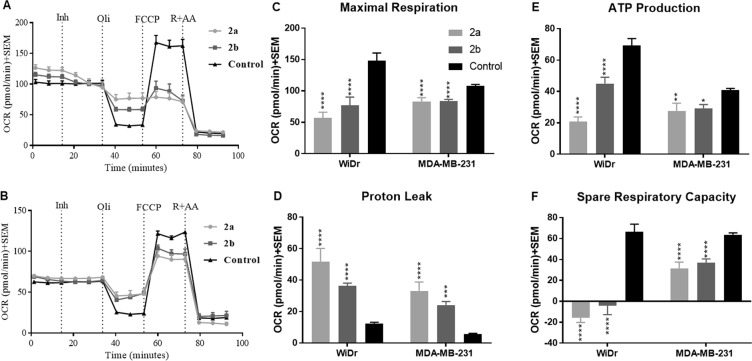
Mitochondrial stress test of compounds **2a** and **2b** at 30 µM. (**A,B**) Represent mitochondrial stress test profile of compounds **2a** and **2b** in (**A**) MCT1 expressing WiDr cell line, and (**B**) MCT4 expressing MDA-MB-231 cell line. Untreated cells were sequentially exposed first to test compound **2a** or **2b** (Inh), followed by oligomycin (Oli), trifluoromethoxy carbonylcyanide phenylhydrazone (FCCP), and rotenone + antimycin a (R + AA). (**C**–**F**) Represent the OCR values of (**C**) maximal respiration, (**D**) proton leak, (**E**) ATP production and (**F**) spare respiratory capacity in WiDr and MDA-MB-231 cell lines. The average + SEM values of three or more individual experiments were calculated. Repeated measures one-way ANOVA was used to calculate statistical significance (*P < 0.05, **P < 0.01, ***P < 0.001, ****P < 0.0001) between test compounds and DMSO control (n = 3).

To assess mitochondrial stress, four parameters including maximal respiration, proton leak, ATP production, and spare respiratory capacity were measured. ATP production via OxPhos is dependent on mitochondrial integrity and can be measured directly via the oxygen consumption rate (OCR). Time-course OCR plots from WiDr and MDA-MB-231 cells were used to calculate **2a** and **2b** treatment-induced changes in specified parameters following the addition of various mitochondrial targeting agents (Fig. 5A,B). Compounds **2a** and **2b** induced significant mitochondrial stress in both WiDr and MDA-MB-231 cell lines. The FCCP induced maximal respiration was significantly decreased in both treated cell lines indicating that compounds **2a** and **2b** inhibit the cells ability to meet increased oxygen demands to retain a mitochondrial proton gradient (Fig. 5C). There was also a significant increase in proton leak, indicative of mitochondrial damage by **2a** and **2b** (Fig. 5D). Furthermore, oxygen consumption for mitochondrial ATP production was significantly decreased in both cell lines (Fig. 5E). Spare respiratory capacity is a value that is not directly linked to mitochondrial damage but represents the cells ability to respond to rapid ATP demands. WiDr and MDA-MB-231 cells exhibited a significant decrease in the spare respiratory capacity in the presence of test compounds (Fig. 5F).

### Fluorescent microscopy

**Figure 6:**
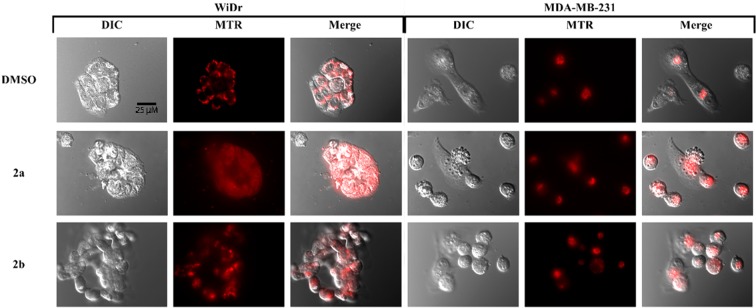
Fluorescent MTR microscopy of (**A**) MDA-MB-231 and (**B**) WiDr cells after 24-hour exposure to 30 μM compound **2a** and **2b**. All images were captured using the same magnification (see scale bar, 25 μm). Note compound **2a** resulted in substantial redistribution of MTR. Images were selected as representative of overall culture appearances.

To evaluate the effects on mitochondrial morphology and vitality, Mitotracker Red CMXROS (MTR) fluorescence imaging experiments were employed. Control WiDr and MDA-MB-231 cultures displayed well-defined MTR-positive mitochondria (Fig. 6). Cells treated with **2a**, however, exhibited a more diffuse and dim general cytoplasmic fluorescence, suggesting loss of MTR from mitochondria. Furthermore, differential interference contrast (DIC) microscope images illustrate substantial membrane blebbing and vesiculation in compound treated cultures (Fig. 6).

### Western blot analysis

**Figure 7:**
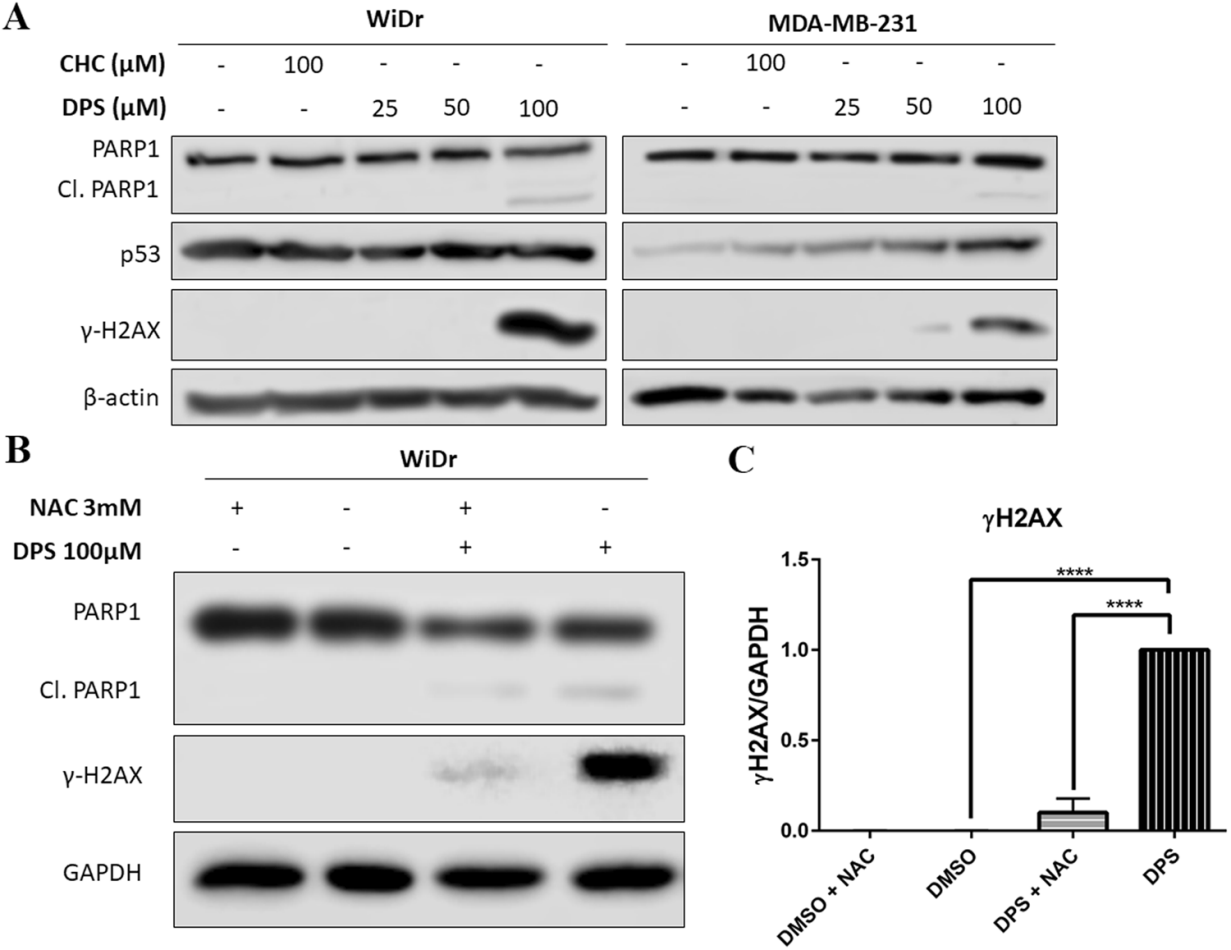
(**A**) Treatment with **2a** (DPS) for 24 hours induced PARP1 cleavage and histone H2AX phosphorylation in WiDr and MDA-MB-231 cells indicative of apoptosis and DNA damage. Further, treatment with **2a** lead to an increase in p53 expression in MDA-MB-231 cells in a dose dependent manner. (**B**) Treatment with the radical scavenger N-acetyl cysteine (NAC) reversed H2AX phosphorylation but not PARP1 cleavage in WiDr cells. (**C**) Densitometry analysis of γ-H2AX when compared to GAPDH as a loading control. Representative western blots of three independent experiments, and are cropped from the full-length images. Full-length blots can be found in the supplementary information, Figs. S2–S4. Repeated measures one-way ANOVA was used to calculate statistical significance (****P < 0.0001).

Owing to the enhanced antiproliferative effects induced by test compound **2a** when compared to CHC, western blot analysis of cell damage and death markers was performed. The master transcription factor p53 regulates the expression of numerous genes involved in cancer progression and is responsible for balancing proliferation and apoptosis^35^. In this regard, we evaluated the effects of compound treatment on p53 expression. Literature reports indicate that MDA-MB-231 and WiDr cells express distinct homozygous mutant forms of p53 that promote an aggressive malignant phenotype with resistance to apoptosis^36,37^. In these studies, we observed that treatment with **2a** induced an increase in p53 expression in MDA-MB-231 cells but not in WiDr (Fig. 7A). Further, PARP1 is involved in the DNA repair pathway through sensing DNA-strand breaks and recruiting repair enzymes^38^. Importantly, PARP1 is a target of caspase cleavage under apoptotic conditions, and the appearance of the PARP1 cleavage product is a widely used indicator of cellular apoptosis. Here, we observed that 100 μM compound **2a** induced PARP1 cleavage in both WiDr and MDA-MB-231 cell lines, strongly suggesting the activation of apoptotic cell death pathways in treated cultures (Fig. 7A). Additionally, DNA damage may be a downstream effect of apoptotic nuclease activity or an increase in reactive oxygen species (ROS) from damaged mitochondria; as illustrated in microscopy and seahorse experiments. Histone 2AX phosphorylation (γH2AX) resulting from DNA single- and double-strand breaks is a widely employed marker for DNA damage^39^. In this regard, we investigated the effect of **2a** on DNA damage marker γH2AX (phosphorylated Ser139, histone H2AX) with a phospho-specific antibody. Treatment with **2a** (100 μM) led to substantial increases in the expression of γH2AX in both WiDr and MDA-MB-231 cell lines (Fig. 7A). Due to the potential of ROS leading to DNA damage and H2AX phosphorylation, we investigated the ability of antioxidant N-acetyl cysteine (NAC) to inhibit DNA damage of compound **2a** as evidenced by H2AX phosphorylation in the highly oxidative cell line WiDr (Fig. 4A). In these studies, we found that **2a-**induced H2AX activation was significantly reduced in cultures treated with NAC (Fig. 7B,C). However, NAC did not reverse PARP1 cleavage in treated cultures (Fig. 7B).

### *In vivo* efficacy studies of MCT1 expressing WiDr tumor xenograft model

Based on *in vitro* cell proliferation inhibition, MCT1 inhibition, and effects on glycolysis/mitochondrial function, we advanced candidate compounds **2a** and **2b** for translational *in vivo* safety and efficacy studies. In this regard we first evaluated the systemic toxicity of candidate compounds in healthy CD-1 mice where it was found that once daily dosage (25 mg/kg) of both **2a** and **2b** were well tolerated over the course of a 16-day treatment period as evidenced by normal body weight gains (Fig. 6A,B) behavior, and grooming patterns. Next, an MCT1 expressing WiDr tumor xenograft model was chosen to evaluate the efficacy of **2a** and **2b**. After tumors were inoculated, they were allowed to reach ~150 mm^3^ volume, and mice were randomly divided into three groups. Group-1 was administered 25 mg/kg (i.p.) of compound **2a**, group-2 was administered with 25 mg/kg (i.p.) of **2b**, and group-3 was designated as control group and given an injection of vehicle. Treatment was continued for 16 days, and tumor volumes were recorded. From this study, there was a significant suppression in tumor volume when treated with TBDPS-CHC derivative **2a (**Fig. 6C,D**)** compared to the control group. During the two-week treatment period, there was no significant loss of body weights in mice between treated and control groups. The Ex-TBDPS-CHC derivative **2b** also suppressed the tumor growth by 28%, but compound **2a** exhibited higher efficacy 36% compared to **2b**. At the end of the study, all the tumors were resected, and the tumor mass was weighed (Fig. 6D).

## Discussion

Altered metabolism is an enabling characteristic of cancer cells that supports tumor growth and progression^2^. Diffusion of metabolites, oxygen, and nutrients throughout the tumor vary and hence, glycolysis and OxPhos machinery exhibit differential expression patterns depending on the microenvironment of the cells^9,10,12^. Interestingly, mitochondrial OxPhos has been linked to proliferation in cancer cells and lactate uptake via MCT1 provides these cells with necessary TCA cycle substrates in support of heightened OxPhos^9,10,12^. Mitochondrial OxPhos is a stable and efficient source of ATP production, but is vulnerable to damage in later stages of cancer progression. Under these conditions, cancer cells exhibit the capability to increase glycolysis to meet the energetic and biosynthetic demands. The evolution of various metabolic phenotypes helps to maintain tumor plasticity and resistance to therapeutic intervention. In this regard, we have evaluated the potential of silicon appended lipophilic CHC analogues **2a-b** to inhibit cancer cell proliferation, MCT1 based lactate uptake, disrupt glycolysis and mitochondrial metabolism, and suppress *in vivo* tumor growth.

Further, the α-cyanocinnamic template has also been shown to inhibit the mitochondrial pyruvate carrier^27^ and hence, improving the lipophilicity of such molecules may lead to disruption of mitochondrial metabolism. Previously it has been shown that synthetic mitochondrial targeted silylated derivatives exhibit potent anti-cancer efficacy and hence gave a rationale to develop lipophilic silylates that may exhibit dual MCT1 and mitochondrial inhibitory properties^40,41^. Silyl ethers **2a** and **2b** derived from CHC with their lipophilic characteristics present an attractive method to increase the cell proliferation. The *in vitro* cell proliferation studies of candidate compounds **2a-b** have shown that these molecules exhibit higher cell proliferation compared to CHC specifically in the MCT1 expressing 4T1 and WiDr cell lines. To evaluate the importance of the silyl group in providing anti-proliferative effects, parent molecules **2a** and **2b** were tested. The non-silylated CHC derivatives **2c** or **2d** did not exhibit any cell proliferation inhibition properties reinforcing the importance of the TBDPS group in providing the cytotoxic properties. To evaluate the MCT1 inhibitory properties of synthesized compounds **2a-d**, we performed a lactate uptake assay as previously described by us^18–20^. Again, both silylated derivatives **2a** and **2b** showed potent MCT 1 inhibition in low µM concentration compared to CHC which typically inhibits MCT1 function at or above 150 µM concentration. The non-silyl derivatives **2c** and **2d** were potent than CHC but much less potent than the silylated derivatives **2a** and **2b**.

The structure of inward-open human MCT1, generated based on our computational studies, appears to be of sufficient quality to identify the binding site of candidate MCT1 inhibitors TBDPS-CHC (**2a**) and Ex-TBDPS-CHC (**2b**). The binding site amino acids determined by the most favorable docking poses of the two compounds is shown to be very similar with the extended **2b** reaching a few additional residues. Surprisingly, many of the analogous amino acids were previously identified by Nancolas *et al*. for AstraZeneca AR-C155858 inhibitor binding to an independently generated rat MCT1 model, also initially modeled in the inward-open state^42^. The concordance of binding site residues, although not fully expected for such a structurally distinct inhibitor, lends confidence in the results obtained here and consistently define the inhibitor binding pocket. The lipophilic phenyl groups of **2a** and **2b** binding to MCT1 is characterized by several hydrophobic contacts, including aromatic stacking of one of the inhibitor phenyl groups to phenylalanine in the binding pocket. The extensive hydrophobic contact surface between the candidate compounds and MCT1 likely leads in large part to the dramatic increase in affinity of these inhibitors over the parent CHC compound. The favorable hydrophobic interactions are supplemented by several putative hydrogen bonds. In fact, it appears most polar atoms in compounds **2a** and **2b** are immediately adjacent to one or more polar side chains, including conserved Tyr34 and Arg313 in human MCT1 (Fig. 3B). The cyano group, or rotationally isosteric carboxyl group, of the derivative compounds specifically interact with the hydroxyl group of conserved Tyr34 and the guanidine group of Arg313 simultaneously, another likely strong contributor to specificity and high affinity of these compounds over parent compound CHC. CHC docking was predicted to be both lower affinity and much lower specificity (based on pose position occupancy). Nonetheless, swapping cyano and carboxyl positions, via bond rotation, maintains hydrogen bonding potential in each case. Some of the uniqueness of **2a** and **2b** inhibitor binding to inward-open MCT1 is likely due to rotational freedom in the inhibitors, exemplified by the imprecise overlay in Fig. 3D. Further, the three-atom insertion to **2b** (C-C-O) increases this rotational freedom and volume making the similarities even more remarkable. The similarity from end to end of the two compounds in the binding pocket may suggest that the three-atom extension is not particularly advantageous. Although, **2b** was estimated to have slightly higher binding affinity than **2a** (−9.8 kcal/mol vs. −9.2 kcal/mol) to MCT1, the number top of poses occupying this site out of 20 was much fewer (5 vs. 11, respectively). This suggests that the specificity of **2b** may be compromised by the three-atom extension, perhaps by the increase in rotational freedom and therefore the entropy of the extended structure. There appears to be some potential for optimization based on the **2a** and **2b** MCT1 docking poses. Of the residues within 4.5 Å of the compounds binding site, the most obvious unsatisfied interaction is that of conserved Glu398, also identified in the binding site of AstraZeneca inhibitor AR-C155858 in rat MCT^42^. Satisfaction of this negative charge with a polar or positively charged substituent might lead to much higher inhibitor affinity and/or specificity albeit with poorer lipid diffusive capacity. In addition, there appears to be an additional hydrophobic pocket near the cyano and carboxyl inhibitor substituents of the inhibitor binding site, currently occupied by the polar end of the candidate compounds. Of course, just outside the 4.5 Å area defining the contact surface for compound **2a** and **2b** there are other potential favorable interactions. The change of the sterically shielded silicon to carbon is not expected to change either the hydrophobic interaction or hydrogen bonding terms. The bond length change could potentially contribute to small differences in calculated binding energies, especially in highly constrained binding pockets - not true in this case (Fig. 3). Further, crystal structure comparison of silicon analogues of known protein agonists show nearly identical binding topologies^43^. Docking poses and calculated energies have also been shown to be very similar for silicon analogues^44^. For these reasons and more practically because Vina is not internally parameterized for Si, we chose to substitute carbon in its place in the current study. There are cases where this would not be advised, namely if the Si is not sterically shielded and/or the binding pocket itself is very conformationally constrained. In the current context, we believe that the computational substitution of carbon for silicon in compounds **2a** and **2b** does not influence the topological outcomes of the top binding poses and contributes minimally to the calculated binding energies. Nonetheless, the newly generated human open-inward MCT1 model may allow us to continue to improve small molecule inhibitor design.

It is interesting to note the possibility of mitochondrial pyruvate carrier (MPC) inhibition with compounds **2a** and **2b**, as inhibition of mitochondrial pyruvate uptake may result in feedback mediated inhibition of extracellular lactate uptake. These observations have been previously reported with similar cyanocinnamic acid compounds^27^, along with aminocarboxy coumarin derivative 7ACC2^45^ as inhibitors of mitochondrial pyruvate uptake and consequent non-binding feedback inhibition of MCT1 mediated lactate uptake. Similarly, effects of compounds **2a** and **2b** on mitochondrial respiration (Fig. 5) may be a result of direct MPC and feedback inhibition of MCT1.

Improved cell proliferation and MCT1 inhibition properties along with lipophilic characteristics prompted us to examine the effects of **2a** and **2b** on glycolysis and mitochondrial OxPhos. To study this, we employed the standard Seahorse XFe96^®^ based glycolysis and mitochondrial stress tests in MCT1 expressing WiDr and MCT4 expressing (MCT1 null) MDA-MB-231. In these experiments, real time measurements of extracellular acidification rate (ECAR) and oxygen consumption rates (OCR) give rise to glycolytic and mitochondrial respiration rates respectively. Changes in ECAR and OCR in cancer cells following the addition of specific metabolites and inhibitors generate a glycolysis and mitochondrial respiratory profile from which metabolic effects of synthesized derivatives can be observed.

In the glycolysis stress tests, the addition of oligomycin eliminates the cells ability to use the mitochondria (OxPhos) as a source of ATP production, and as a result cells reach a maximum theoretical glycolysis or referred to as glycolytic capacity. With exposure to the compounds, glycolytic capacity remained unchanged in WiDr, and there was a significant reduction in MDA-MB-231. This observation may offer evidence that the effectiveness of the compounds on glycolysis may depend on the intrinsic metabolic phenotype of the cell, as glycolysis is upregulated in the MDA-MB-231 cell line (Fig. 4). Further, it was interesting to note that both compounds resulted in a stimulated rate of glycolysis in the WiDr cell line. This data suggests that mitochondrial disruption caused by compound treatment may stimulate a heightened level of glycolysis to keep up with energy demands. This data further bolsters the claim that the metabolic effects of our compounds are a function of basal metabolic phenotype (Fig. 4). These results translate to glycolytic reserve which is the ability of the cell to switch its metabolic dependencies to glycolysis for ATP production. In both cell lines this parameter is significantly reduced especially with exposure to the directly attached silyl ether **2a**.

The mitochondrial stress test with test compounds has shown potent inhibition of numerous parameters of mitochondrial respiration. Exposure of cells to mitochondrial membrane proton uncoupler FCCP stimulates cells to increase oxidation of TCA cycle substrates and oxygen consumption to maximum capacity to replenish the proton gradient, and the difference between basal respiration and maximal respiration describes the spare respiratory capacity of a given cell line. Further, mitochondrial ATP production can be evaluated by observing the change in OCR after addition of ATP synthase inhibitor oligomycin. Mitochondrial proton leak due to compound induced damage to the mitochondrial membrane can also be evaluated from the mitochondrial respiratory profile. Here, the presence of test compounds potently inhibited maximal respiration, mitochondrial ATP production, and spare respiratory capacity, and lead to an increase in proton leak. Interestingly, there was a more significant effect on mitochondrial respiration in the more oxidative WiDr cell line (Fig. 5).

To visualize compound effects on mitochondrial morphology and vitality, mitotracker red CMXROS (MTR), a fluorescent mitochondrial probe that binds proportional to membrane potential, was used. In these studies, untreated cells exhibited a stable MTR intensity and structural organization of the mitochondria were easily distinguishable. After treatment with compound **2a**, cultures exhibited a diffuse mitochondrial localization when compared to control cultures. The diffusion of mitochondria is suggestive of mitochondrial damage caused by our compound as the compartmentalization of mitochondria is found in healthy cells with functioning mitochondria. Also, it is quite possible that mitochondrial damage induced by compound **2a** lead to MTR to leakage, resulting in an apparent diffused mitochondrial morphology. Regardless, mitochondria damage was apparent following compound treatment.

**Figure 8:**
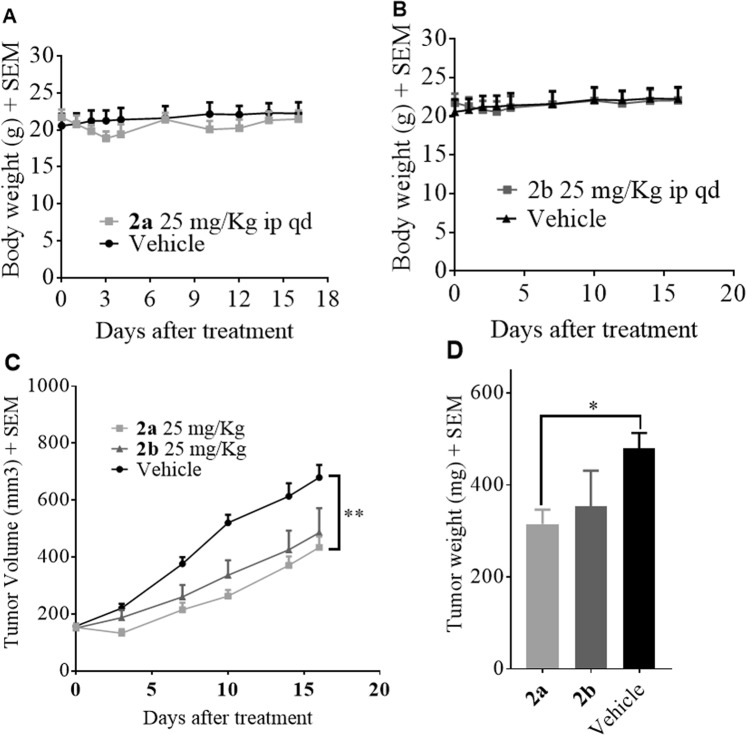
*In vivo* safety and efficacy study of lead compounds **2a** and **2b**. Systemic toxicity study of compounds (**A**) **2a** and (**B**) **2b** in CD-1 mice (n = 6). Note normal body weight changes in treated mice when compared to vehicle control group. (**C**) Tumor volumes and (**D**) tumor mass of WiDr tumor xenograft study of **2a** and **2b** in athymic nude mice (n = 6). Mann-Whitney’s test was performed to calculate statistical significance for this study (*P < 0.05, **P < 0.01).

The connection between cellular metabolism and proliferation is a complex interaction that is strongly influenced by an elaborate relationship between the metabolic and cell cycle machinery^46–48^. In each phase of the cell cycle there are checkpoints on both biosynthetic and bio-energetic supply that are required to maintain proliferation^46–48^. The inhibition of metabolic processes can interrupt cell proliferation and result in programmed cell death. In our study it was found that exposure to our compound caused PARP cleavage in both cell lines and an increase in the p53 marker. It is possible that an increase in mutant p53 cell lines and concomitant PARP1 cleavage at 100 μM **2a** may suggest regained pro-apoptotic functions of p53 as observed with other literature reported experimental anticancer drugs^49^. Further, N-acetyl cysteine was able to reverse compound induced DNA damage as indicated by reduced H2AX phosphorylation – indicating that increased ROS in treated cultures was likely responsible for DNA damage. This observation further supports mitochondrial targeting with compound **2a** as dysfunction of the mitochondria can result in release of ROS. However, we did not observe a noticeable decrease in PARP1 cleavage with the treatment of NAC, suggesting that apoptotic events caused by **2a** treatment may be triggered independently of ROS induced DNA damage. In this regard, it is likely that compounds **2a** and **2b** act through numerous mechanisms to illicit anticancer efficacy observed in WiDr xenograft study (Fig. 8B,C), which may qualify these candidates for combination studies with clinically used anticancer agents to realize their full potential.

In conclusion, novel silyl cyanocinnamic acid derivatives have been synthesized and evaluated as potential anticancer agents. *In vitro* studies demonstrated that lead compounds **2a** and **2b** exhibited high cancer cell proliferation inhibition and potent MCT1 inhibition properties as determined by MTT assay and lactate-uptake studies, respectively. *In vitro* effects on glycolysis and mitochondrial metabolism illustrated that the **2a** and **2b** significantly perturbed both metabolic pathways. *In vivo* studies demonstrated that candidate compounds were well tolerated in healthy mice and exhibited good single agent tumor growth inhibition properties in a WiDr colorectal cancer xenograft model.

## Materials and Methods

### Cell lines and culture conditions

Human triple-negative breast cancer MDA-MB-231 cells (ATCC) were grown in DMEM supplemented with 10% FBS (Atlanta Biologicals) and penicillin-streptomycin (50U/ml, 50 µg/ml, Invitrogen). Human colorectal adenocarcinoma WiDr cells (ATCC) were cultured in MEM medium supplemented with 10% FBS and penicillin-streptomycin. Although WiDr was deposited with the ATCC as a colon adenocarcinoma line established from a 78-year-old female, DNA fingerprinting has shown this line to be a derivative of HT-29^50^. Murine metastatic breast cancer 4T1 cells were grown in RPMI-1640 supplemented with 10% FBS and penicillin-streptomycin. Human estrogen receptor positive breast cancer MCF7 cells (Masonic Cancer Center, University of Minnesota) were grown in MEM supplemented with 6% FBS, EGF (0.01 µg/ml), NEAA, HDC (1 µg/ml), insulin (10 µg/ml), HEPES (12 mM) and sodium pyruvate (1 mM). Rat brain endothelial 4 (RBE4, a gift from F. Roux^18^) cells utilized in MCT1 inhibition assay were cultured in 1:1 α-MEM and F-10 HAM supplemented with 10% FBS (heat inactivated), 1 ng/ml basic fibroblast growth factor, 0.3 mg/ml geneticin, 1% antibiotic-antimycotic.

### MCT1 inhibition assay via L-[^14^C]-lactate uptake in RBE4 cells

MCT1 inhibition study was carried out using rat brain endothelial cells (RBE4) as reported previously^18^.

### MTT based cell proliferation inhibition assay

Cell proliferation inhibition was evaluated using 3-(4,5-dimethylthiazol-2-yl)-2,5-diphenyl tetrazolium bromide (MTT) assay as reported previously^19^.

### Homology modeling of human MCT1 protein structure

Human MCT1 membrane protein structure was generated by homology modeling with MODELLER 9.18 using the inward-open human glucose transporter 1 (hGLUT1) as a structural template, PDB file: 5eqi^31,32^. Due to the minimal sequence similarity between the MCT1 and hGLUT1, we generated a final template alignment by consensus sequence alignment guided by consensus transmembrane spanning domain prediction. The alignments were generated using the following sequences: Human MCTs 1, 2, 3, 4, and 6; Human GLUTs 1 and 3; Bovine GLUT 5; Rat GLUT 3; and with and without *E. coli* major facilitator superfamily proteins LacY, EmrD and GlpT. Alignment programs PROMALS, MAFFT and MSAProbs were used to align the sequences followed by manual adjustment to eliminate gaps in the transmembrane spanning domains by alignment consensus and transmembrane domain consensus prediction^51–53^. Human MCT1 sequence similarity to human GLUT1 was 29% while similarity to *E. coli* LacY and GlpT were 24% and 23%, respectively. Transmembrane domain prediction software TMHMM, PloyPhobius, MEMSAT and CCTOP were used to generate a consensus membrane spanning topology^54–57^. The adjusted sequence alignment was used as input into MODELLER 9.18 to build the MCT1 structure with the last 50 C-terminal amino acids removed. These amino acids are not represented in the template structure, are not considered to be important for inhibitor binding and are not part of a transmembrane spanning domain. Five independent models of MCT1 were built from the hGLUT1 structure template using default optimization and molecular dynamics refinement. An evaluation of structure fitness was based on the intrinsic discrete optimized protein energy score (DOPE) of MODELLER and an evaluation of charged residue rotamer orientation in the transmembrane spans, i.e. avoiding exposure of charged residues to the putative lipid bilayer. One model of human MCT1 was used for subsequent analysis. As with the homology model of rat MCT1 previously built by Manoharan, *et al*., we consider the models synthesized to be of intermediate quality but predictive in nature^33^. We briefly compare binding site residues identified here with those identified by Nancolas, *et al*. for rat inhibitor binding^42^.

### Molecular docking studies

Human MCT1 homology model was used for computational docking studies of parent compound CHC and the TBDPS-CHC and Ex-TBDPS-CHC derivatives. Autodock Vina was used to dock the MCT inhibitors to the inward open homology model^34^. The intrinsic silicon atom of the two derivatives was changed *in-silico* to carbon for compatibility with the docking software. Partial charges were assigned to each atom of the inhibitors by the antechamber program built into UCSF Chimera using the AM1-BCC model^58^. The inhibitor docking search area was set intentionally broad, encompassing the entire membrane spanning domain and extending several angstroms toward the inward-open aqueous surface. Other variables such as adding hydrogens to the protein and removing non-polar hydrogens from the inhibitors were set to default values in Autodock Vina. Two independent docking calculations for each inhibitor were performed on human MCT1. Each docking run returned the top ten inhibitor poses determined by estimating an energy of interaction or binding affinity. Docked inhibitor poses were viewed with UCSF Chimera and the top poses were selected for further analysis. All MCT1 residues within 4.5 Å of the docked inhibitors were determined and compared for each compound. Autodock Vina estimated individual binding affinities were compared. Further, the number of poses nearly identical ( < 2 Å RMSD) to the most favorable docked pose, estimated by position, orientation and MCT1 residues contacted for each inhibitor was used as a surrogate for binding specificity.

### Seahorse XFe96^®^ assessment of glycolysis and mitochondrial respiration

20,000 cells/well were plated in 96-well Seahorse plates (Agilent, part no. 101085-004) and incubated 16–24 hours at 37 °C at 5% CO_2_. Flux pack sensors (Agilent, part no. 102416-100) were hydrated with XF calibrant solution (Agilent, part no. 100840-000) overnight at 37 °C in a non-CO_2_ incubator. The serum free assay media was prepared from Seahorse base medium (Agilent, part no. 102353-100) enriched with glutamine (1 mM) and sodium pyruvate (1 mM). The pH of the media was adjusted to 7.4. For glycolysis stress test the serum free assay media was used. For the mitochondrial stress test glucose (10 mM) was added to make an enriched serum free assay media. For both stress tests an 8X stock concentration of test compounds in their respective media was prepared for microplate injections in port A. Stock solutions of glucose (90 mM), oligomycin (10 μM), and 2-deoxyglucose (Chem Impex, 550 mM) were prepared such that their final working concentrations are 10 mM, 1 μM and 50 mM, respectively, for glycolysis stress test. For mitochondrial stress test, stock solutions of oligomycin (9 µM), FCCP (2.5–10 µM, cell line dependent), rotenone + antimycin A (5.5 µM) were prepared such that their final concentrations were 1 µM, 0.25–1 µM, and 0.5 µM, respectively in the enriched test media. Under glycolytic stress test, the cells were treated with test compounds, followed by the addition of glucose, oligomycin and 2-deoxyglucose at 14.29, 33.8, 53.35, 72.87 minutes, respectively. Under mitochondrial stress test, cells were treated with test compounds, followed by the addition of oligomycin, FCCP, and rotenone + antimycin A, at 14.29, 33.8, 53.35, 72.87 minutes, respectively. Extracellular acidification rates (ECAR) and oxygen consumption rates (OCR) were recorded in real-time for glycolysis and mitochondrial stress tests, respectively, using a Seahorse XFe96^*®*^ analyzer (Agilent). The parameters related to glycolytic and mitochondrial functions were calculated utilizing the Wave 2.4.0 software (Agilent). ATP rate assay experiment was performed using manufactures (Agilent) protocol, and respective mitochondrial and glycolytic ATP rates were normalized to protein (BCA assay) and calculated using Wave 2.4.0 software.

### Fluorescent microscopy studies

MDA-MB-231 or WiDr cells (5 × 10^4^ cells/mL) were seeded in glass-bottom dishes (MatTek Corp, part no. P35G010C) and incubated for 48 hours. Test compound (30 μM) was added and cells were again incubated for 24 hours prior to fluorescent microscopic imaging. In some cultures, MitoTracker Red CMXROS (Invitrogen, M7512, 100 nM) was added 15 min prior to imaging. The growth media was removed and replaced with 5% FBS in 1X PBS for imaging. Cells were then examined and photographed using a Nikon TE2000 epifluorescent microscope and camera. The images shown are representative of at least 3 fields of view of three separate experiments.

### Western blot analysis

50,000 WiDr or MDA-MB-231 cells were seeded in 100 mm dishes and were incubated for 48 hours. Cells were then treated with test compounds for 24 hours, washed twice with 1X PBS, solubilized in 200 μL SDS boiling buffer (5% w/v SDS, 10% v/v glycerol, and 60 mM Tris pH 6.8), and sonicated. The resulting cell lysate was then assayed for protein using the Pierce BCA protocol. A volume of test sample containing 10 μg protein was loaded on SDS PAGE gel for electrophoresis according to manufacturer’s instructions. Proteins were transferred from the gel to nitrocellulose membrane according to the manufacturer’s instructions. Membranes were blocked for 1 hr at 35 °C using 10% (w/v) non-fat milk in PBST and were exposed to primary antibody. PARP1 (rabbit polyclonal IgG, Origene, TA321555), p53 (rabbit polyclonal IgG, Santa Cruz, sc50329), and γ-H2AX (Upstate 05-636, 1:2500) were detected and visualized using HRP chemiluminescence. For relative quantitation, β-actin (mouse monoclonal IgG C4, Millipore, MAB1501, 1:10,000) and GAPDH (mouse monoclonal IgG, Santa Cruz, sc47724, 1:100) were detected and measured as a control protein.

### *In vivo* systemic toxicity study

Five-week-old healthy CD-1 mice (Charles River) were obtained and acclimatized for one week prior to treatment. Mice (n = 6) were grouped randomly based on average body weight. Group-1 was administered with compound **2a** or **2b** (20 mg/kg, ip, qd) and group-2 was administered with vehicle (10% DMSO, 10% PEG, 40% HS-15 solution (18.8%w/v) ip, qd) intraperitoneally once daily, six days a week for a total of 16 days of treatment. Mice body weights were recorded daily as a proxy for animal health and were also examined for proper activity and grooming patterns. At the end of the study, mice were euthanized. A graph of days of treatment versus body weight ± SEM was generated using GraphPad software. All procedures performed using this method were approved by the University of Minnesota Institutional Animal Care and Use Committee (IACUC, protocol no. 1611-34326A) and are in accordance with all guidelines and regulations.

### *In vivo* tumor growth inhibition study

WiDr cells (5 × 10^6^ cells) were suspended in 1:1 mixture of matrigel (Corning, cat. no. 356237) and PBS and injected subcutaneously into the right flank of female athymic nude mice (Charles River). The mice were randomly assigned into 3 groups (n = 6 mice per group). Vertical and horizontal diameters of tumors were measured every two- or three-days using calipers. The tumor volumes were calculated assuming a perfect sphere using the formula V = ab^2^/2 where ‘a’ is the longer diameter of the tumor and ‘b’ is the shorter diameter of the tumor. Treatment was initiated when the tumor volume reached 100 mm^3^. The study was terminated after sixteen days and tumors were then resected and weighed. All procedures performed using this method were approved by the University of Minnesota Institutional Animal Care and Use Committee (IACUC, protocol no. 1612-34444A) and are in accordance with all guidelines and regulations.

### Ethical statement

The animal studies were approved and conducted consistent with University of Minnesota IACUC protocols 1611-34326A (systemic toxicity study Fig. 8A,B) and 1612-34444A (*in vivo* efficacy study Fig. 8C,D).

### Statistical analysis

Statistics were computed using GraphPad Prism version 7.0. Repeated measures one-way ANOVA was used for *in vitro* studies and Mann-Whitney test was used for *in vivo* studies. A *P*-value of <0.05 was considered significant where *P < 0.05; **P < 0.01; ***P < 0.001; ****P < 0.0001.

## Supplementary information

Development of Novel Silyl Cyanocinnamic Acid Derivatives as Metabolic Plasticity Inhibitors for Cancer Treatment
