# Supplementary material for: Development of Novel Silyl Cyanocinnamic Acid Derivatives as Metabolic Plasticity Inhibitors for Cancer Treatment
Source: Sci Rep. 2019 Dec 4;9:18266. doi: 10.1038/s41598-019-54709-7 (PMC6892925; doi:10.1038/s41598-019-54709-7)
Supplement: Supplementary file 1 — Development of Novel Silyl Cyanocinnamic Acid Derivatives as Metabolic Plasticity Inhibitors for Cancer Treatment [file 41598_2019_54709_MOESM1_ESM.pdf]

Supplementary Information

Development of Novel Silyl Cyanocinnamic Acid Derivatives as Metabolic Plasticity Inhibitors for Cancer Treatment

Grady L. Nelson[a], *Conor T. Ronayne*[a], *Lucas N. Solano*[a], *Sravan K. Jonnalagadda*[a], *Shirisha Jonnalagadda*[a], *Jon Rumbley*[a,d], *Jon Holy*[a,c], *Teresa Rose-Hellekant*[a,c], *Lester R. Drewes*[a,c], *Venkatram R. Mereddy*[a,b,d*]

[a]Integrated Biosciences Graduate Program, University of Minnesota, Duluth, MN 55812
[b]Department of Chemistry and Biochemistry, University of Minnesota Duluth, Duluth, MN 55812
[c]Department of Biomedical Sciences, Medical School Duluth, University of Minnesota, MN 55812
[d]Department of Pharmacy Practice & Pharmaceutical Sciences, University of Minnesota, Duluth, MN 55812

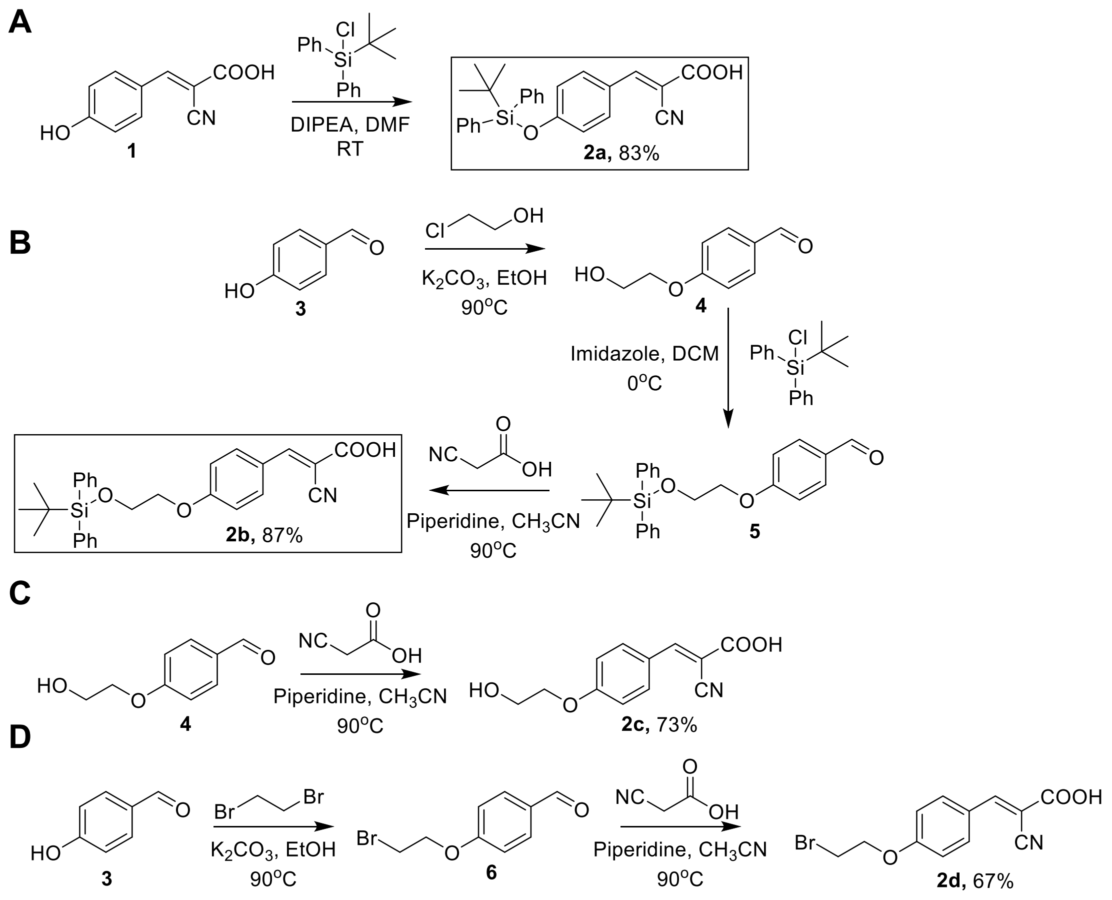

Supplemental Figure 1: Synthetic scheme of silylated CHC analogs (**A**) **2a** and (**B**) **2b** and non-silylated analogs (**C**) **2c** and (**D**) **2d**.

Synthesis of directly attached TBDPS-CHC **2a** was accomplished by reacting commercially available CHC **1** with TBDPS-Cl in the presence of DIPEA. Upon completion, the reaction mixture was poured over 3N HCl and the resulting precipitate was filtered and washed with hexanes to obtain pure **2a** (Fig 1A). Synthesis of Ex-TBDPS-CHC **2b** was achieved first by reacting *p*-hydroxybenzaldehyde **3** with chloroethanol under basic and heated conditions to obtain the intermediate *p*-hydroxyethylbenzaldehyde **4**, which was subsequently reacted with TBDPS-Cl in the presence of imidazole. The resulting TBDPS substituted aldehyde **5** was condensed with cyanoacetic acid in the presence of piperidine and refluxed in acetonitrile for 10 hours. Upon completion of the reaction, the solution was poured over ice and 3N HCl. The resulting precipitate was then filtered and washed with diethyl ether to obtain pure **2b** (Fig 1B). To demonstrate the importance of the silyl group in providing biological activity, compound **2c** and **2d** were synthesized. Specifically, compound **2d** was synthesized as a homolog of **2b** to demonstrate that hydrophobicity offered by TBDPS template is necessary for biological activity. Similarly, the products of hydroxyethyl-CHC **2c** and bromoethyl-CHC **2d** were obtained via a standard Knovenagel condensation of the corresponding aldehydes (**4** and **6**, respectively) with cyanoacetic acid in the presence of piperidine (Fig 1C & 1D).

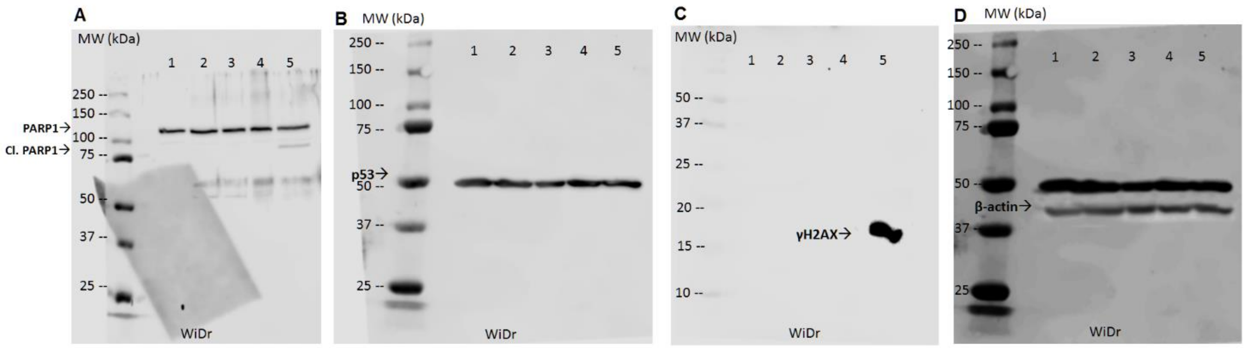

Supplemental Figure 2: Full-length blots of cropped images depicted in manuscript Figure 7A. Lanes labeled 1-5 indicate whole-cell lysates of WiDr cells with the following treatments: (1) DMSO, (2) cyanohydroxycinnamic acid (CHC, 100µM), (3) **2a** (25µM), (4) **2a** (50µM), (5) **2a** (50µM). (**A**) WiDr whole-cell lysates resolved on an 8% SDS-PAGE gel and probed for PARP-1. (**B**) WiDr whole-cell lysates resolved on an 8% SDS-PAGE gel and probed for p53. (**C**) Protein from WiDr whole-cell lysates resolved on a 12% SDS-PAGE gel and probed for γH2AX. (**D**) WiDr whole-cell lysates resolved on an 8% SDS-PAGE gel and probed for β-actin and p53 (note arrow indicating β-actin band used in Figure 7A). Due to saturation of p53 band, an independent exposure was performed, as illustrated in panel B, above. Please refer to methods section for details.

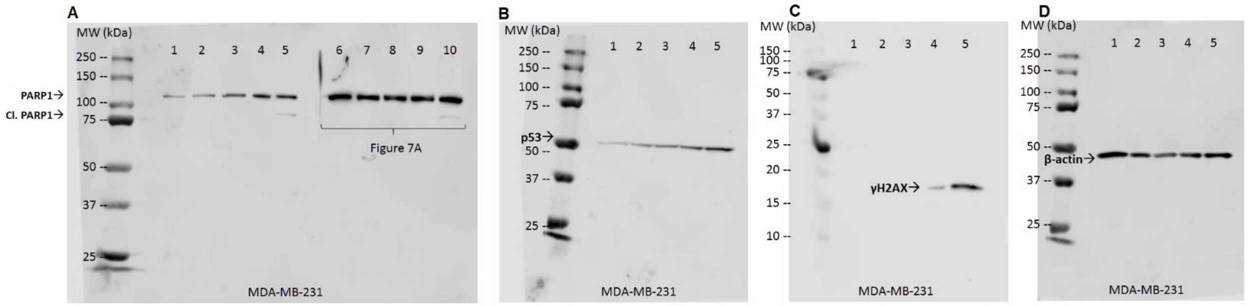

Supplemental Figure 3: Full-length blots of cropped images depicted in manuscript Figure 7A. Lanes labeled 1-5 indicate whole-cell lysates of MDA-MB-231 cells with the following treatments: (1) DMSO, (2) cyanohydroxycinnamic acid (CHC, 100µM), (3) **2a** (25µM), (4) **2a** (50µM), (5) **2a** (50µM). (**A**) MDA-MB-231 whole-cell lysates resolved on an 8% SDS-PAGE gel and probed for PARP-1. Note samples 6-10 indicate the same treatment as 1-5 above, but from independent replicates. PARP-1 from samples 6-10 were used in Figure 7A, as noted. (**B**) MDA-MB-231 whole-cell lysates resolved on an 8% SDS-PAGE gel and probed for p53. (**C**) Protein from MDA-MB-231 whole-cell lysates resolved on a 12% SDS-PAGE gel and probed for γH2AX. (**D**) MDA-MB-231 whole-cell lysates resolved on an 8% SDS-PAGE gel and probed for β-actin. Please refer to methods section for details.

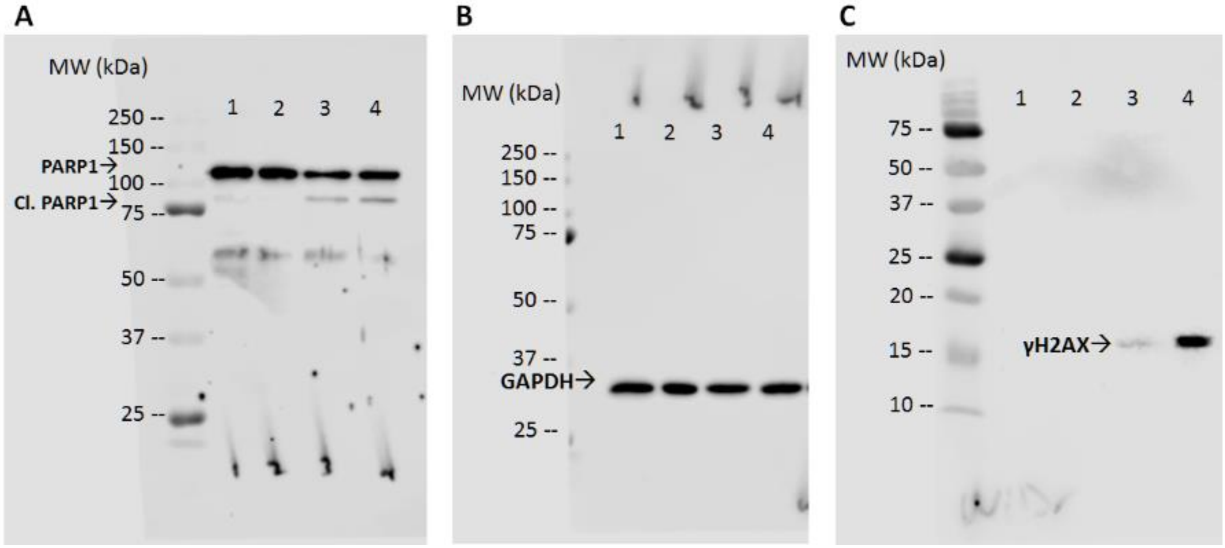

Supplemental Figure 4: Full-length blots of cropped images depicted in manuscript Figure 7B. Lanes labeled 1-4 indicate whole-cell lysates of WiDr cells with the following treatments: (1) DMSO + N-Acetyl cysteine (NAC), (2) DMSO, (3) **2a** (100μM) + NAC, (4) **2a** (100μM). (**A**) Protein from WiDr whole-cell lysates resolved on an 8% SDS-PAGE gel and probed for PARP-1. (**B**) Protein from WiDr whole-cell lysates resolved on an 8% SDS-PAGE gel and probed for GAPDH. (**C**) Protein from WiDr whole-cell lysates resolved on a 12% SDS-PAGE gel and probed for γH2AX. Please refer to methods section for details.

Spectral Characterization

(E)-3-(4-((tert-butyldiphenylsilyl)oxy)phenyl)-2-cyanoacrylic acid (***2a***)

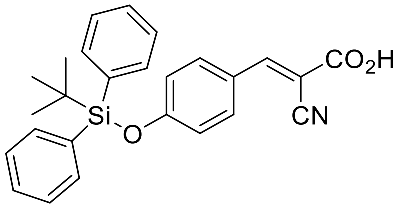

^{1}H NMR (500 MHz, $CDCl_3$):

δ ppm 8.15 (s, 1H), 7.84 (d, J = 9 Hz, 2H), 7.67 - 7.72 (m, 4H), 7.43 - 7.50 (m, 2H), 7.37 - 7.43 (m, 4H), 6.86 (d, J = 9 Hz, 2H), 1.12 (s, 9H)

^{13}C NMR (126 MHz, $CDCl_3$):

δ ppm 168.0, 161.1, 156.1, 135.3, 133.9, 131.7, 130.4, 128.0, 124.4, 120.8, 115.6, 98.2, 26.4, 19.5

HRMS (ESI) m/z:

calculated for $C_{17}H_{22}N_2O_3$ $[M+Na]^+$: 428.168

found 428.146

Elemental Analysis C, H, N

Anal. Calcd for $C_{26}H_{25}NO_3Si$ (427.16): C 73.04, H 5.89, N 3.28.

Found: C 72.34, H 5.96, N 3.20

(E)-3-(4-(2-((tert-butyldiphenylsilyl)oxy)ethoxy)phenyl)-2-cyanoacrylic acid (***2b***):

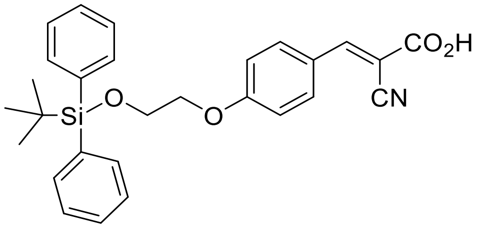

^{1}H NMR (500 MHz, $CDCl_3$) δ ppm 8.24 (br. s., 1H), 8.01 (d, J = 8 Hz, 2H), 7.67 - 7.74 (m, 4H), 7.36 - 7.48 (m, 6H), 6.94 (d, J = 8 Hz, 2H), 4.16 (t, J = 5 Hz, 2H), 4.02 (t, J = 5 Hz, 2H), 1.07 (s, 9H).

^{13}C NMR (126 MHz, $CDCl_3$) δ ppm 168.8, 163.8, 156.1, 135.6, 134.2, 133.3, 129.9, 127.8, 124.1, 115.4, 98.4, 69.4, 62.3, 26.8, 19.2

Elemental Analysis C, H, N

Anal. Calculated for $C_{28}H_{29}NO_4Si\ Na^+$ (494.18): C 67.99, H 5.91, N 2.83.

Found: C 69.06, H 5.98, N 2.90.

E)-2-cyano-3-(4-(2-hydroxyethoxy)phenyl)acrylic acid (***2c***):

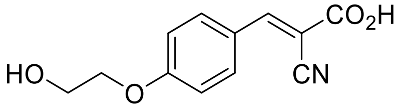

^{1}H NMR (500 MHz, MeOH-d_6):

δ 8.09 (s, 1H), 7.98 (d, J = 9 Hz, 2H), 7.12 (d, 2H), 4.20 (t, 2H), 3.97 (t, J = 5 Hz, 2H)

^{13}C NMR (126MHz, MeOH-d_6):

δ 167.5, 161.8, 149.7, 131.8, 125.4, 118.4, 114.6, 107.0, 69.4, 60.1.

*(E)-3-(4-(2-bromoethoxy)phenyl)-2-cyanoacrylic acid (**2d**)*

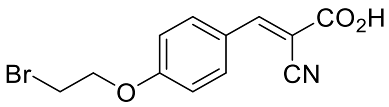

¹H NMR (500 MHz, $CDCl_3$) d 9.87 (s, 1H), 7.82 (d, J = 8.79 Hz, 2H), 6.99 (d, J = 8.79 Hz, 2H), 4.35 (t, J = 6.10 Hz, 2H), 3.56 - 3.76 (m, 2H)

¹³C NMR (126 MHz, $CDCl_3$) d 164.7, 161.9, 153.8, 133.4, 125.1, 116.4, 115.2, 100.8, 67.9, 28.5

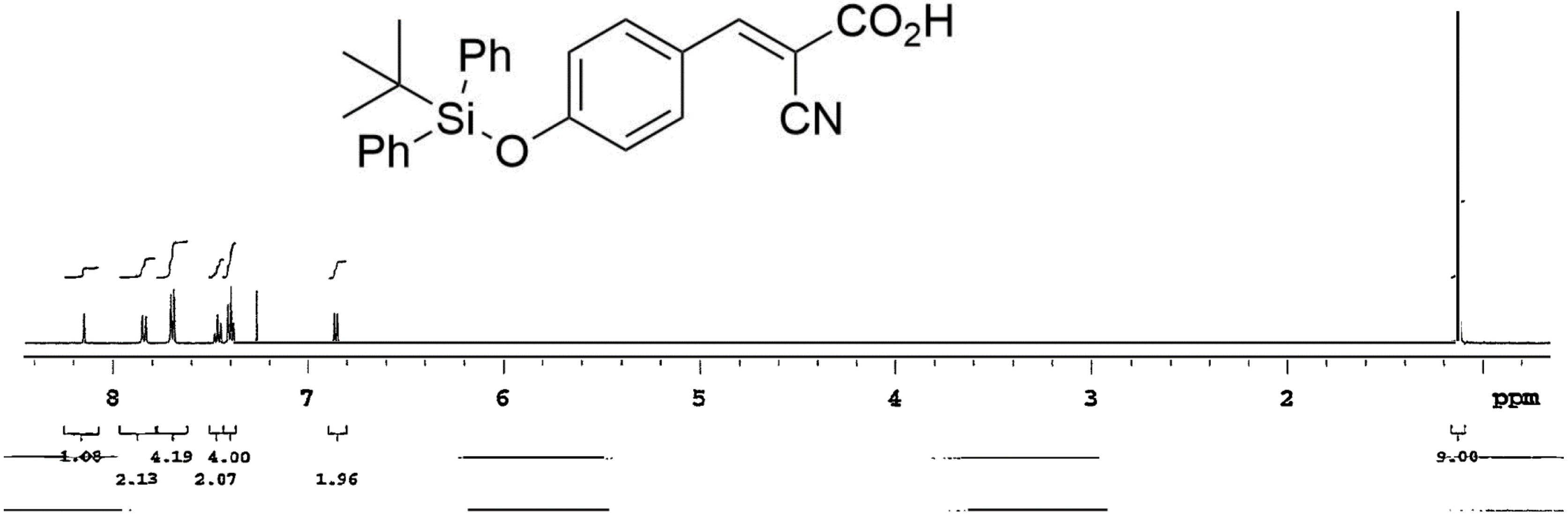

Ph
Si
Ph
O
CO2H
CN
8
7
6
5
4
3
2
ppm
1.08
2.13
4.19
2.07
4.00
1.96
9.00

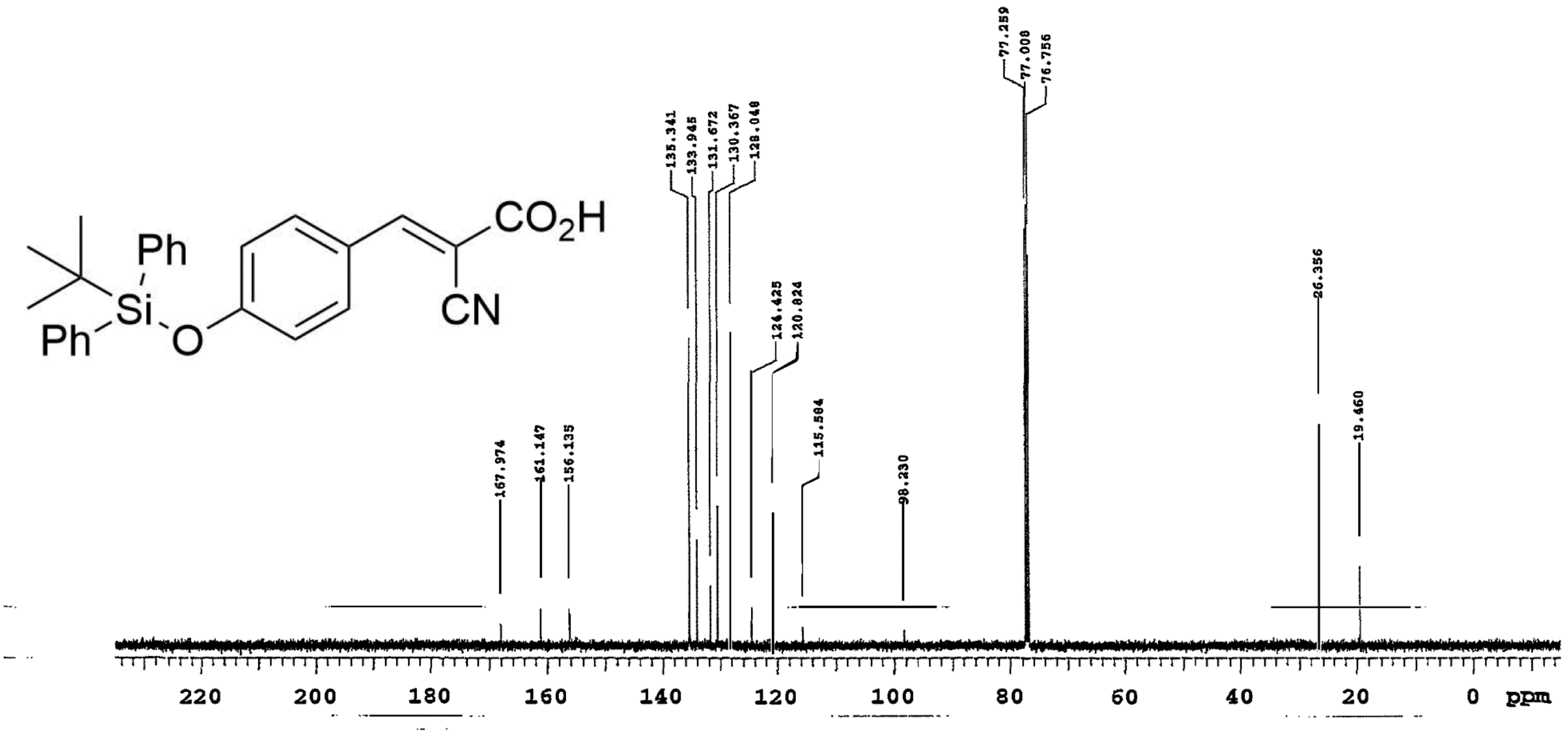
Ph
Si
Ph
O
CO2H
CN
167.974
161.147
156.135
135.341
133.945
131.672
130.367
128.048
124.425
120.824
115.584
98.230
77.259
77.008
76.756
26.356
19.460
220
200
180
160
140
120
100
80
60
40
20
0
ppm

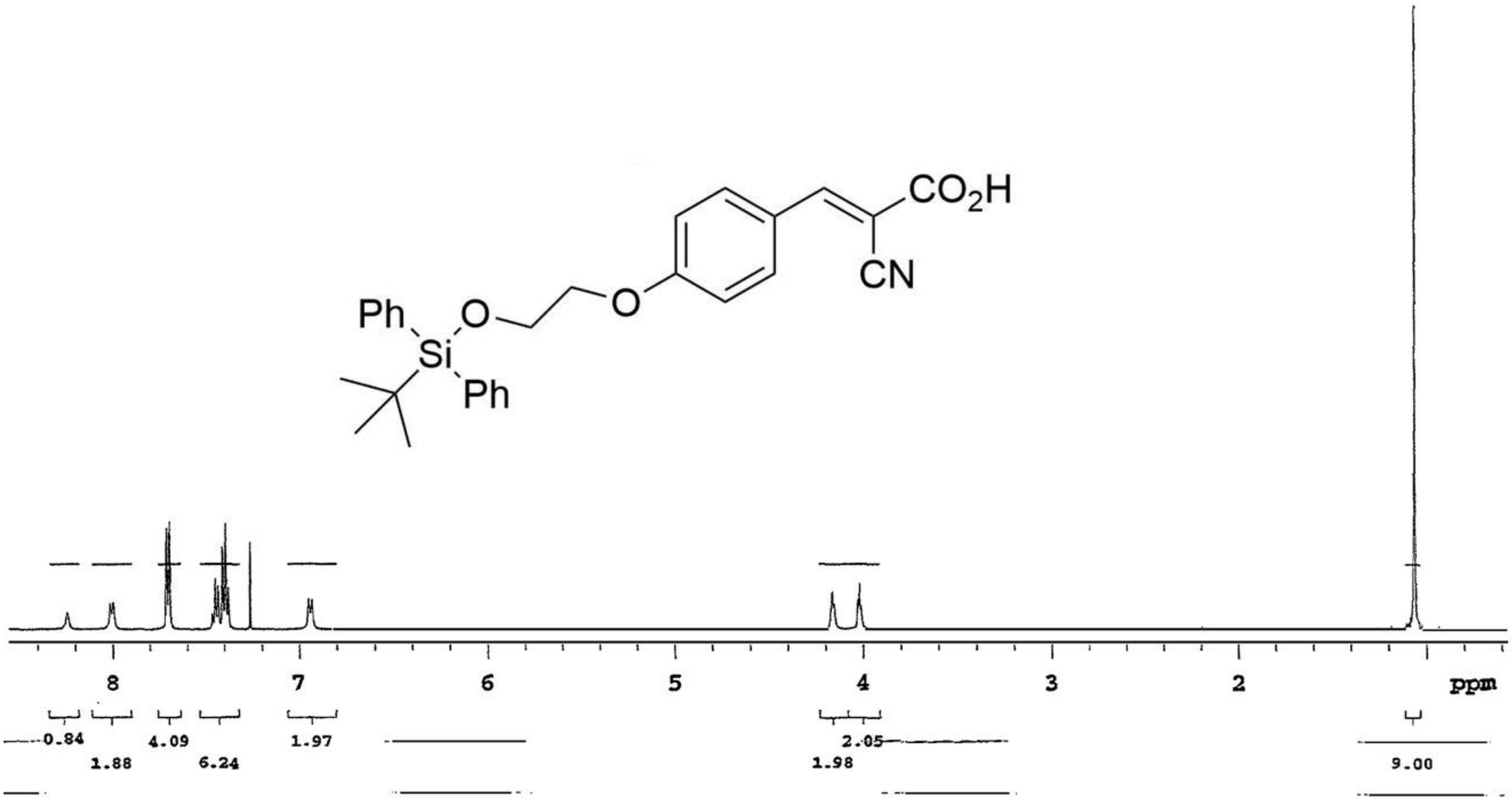

Ph
O
Si
Ph
O
CO2H
CN
8
7
6
5
4
3
2
ppm
0.84
1.88
4.09
6.24
1.97
2.05
1.98
9.00

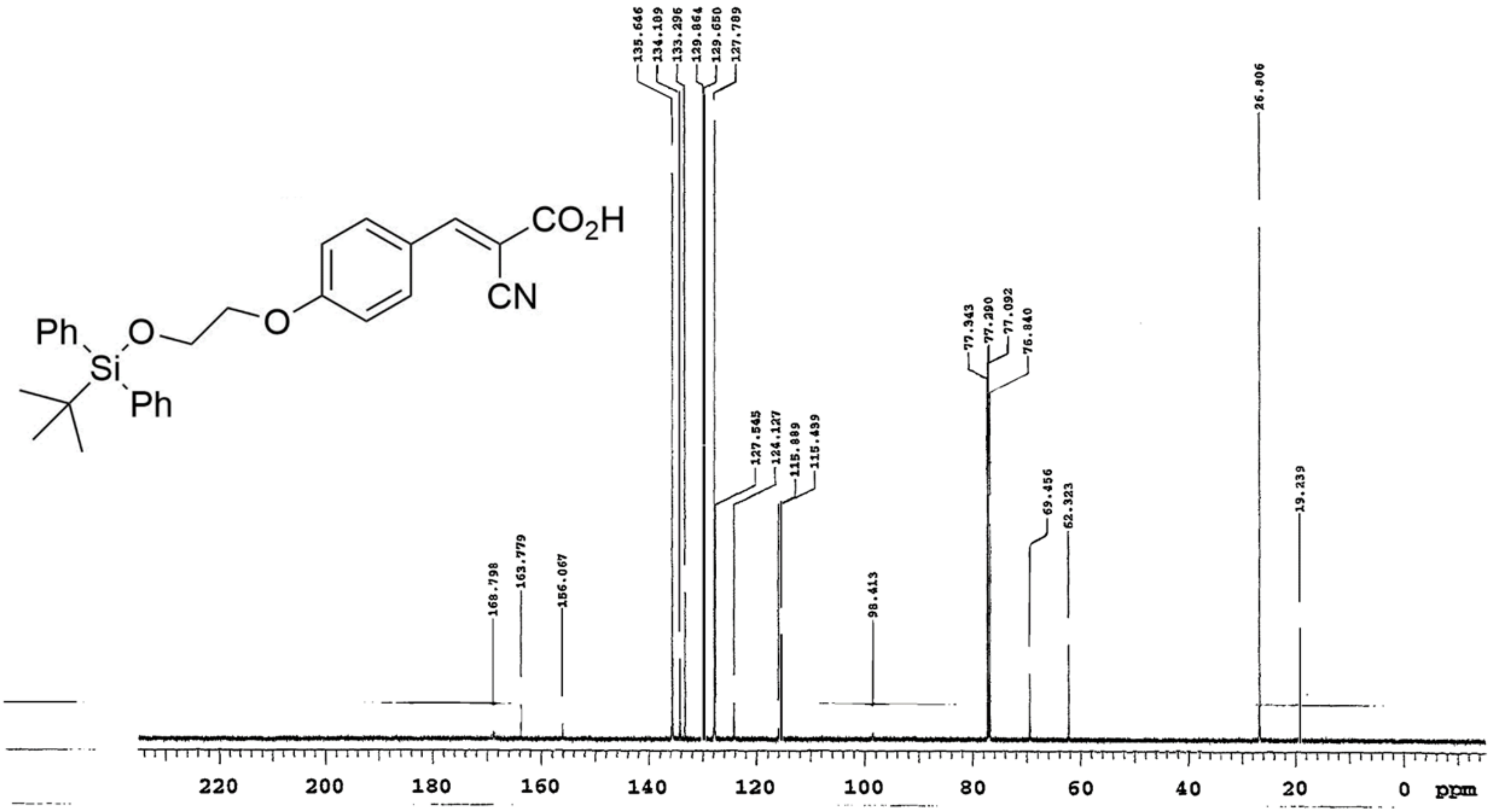

Ph
Si
Ph
O
O
CO2H
CN
135.646
134.189
133.296
129.864
129.650
127.789
127.545
124.127
115.889
115.439
168.798
163.779
156.067
98.413
77.343
77.290
77.092
76.840
69.456
62.323
26.806
19.239
220
200
180
160
140
120
100
80
60
40
20
0
ppm

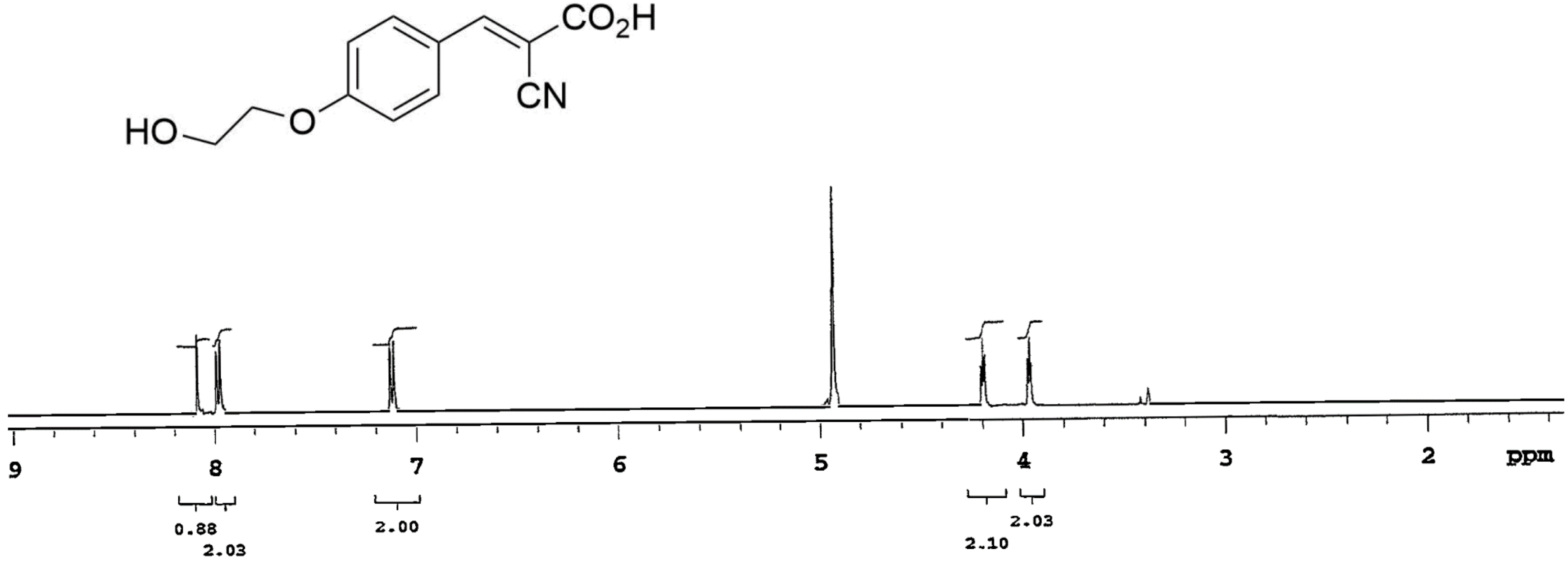

HO
O
CO2H
CN
9
8
7
6
5
4
3
2
ppm
0.88
2.03
2.00
2.10
2.03

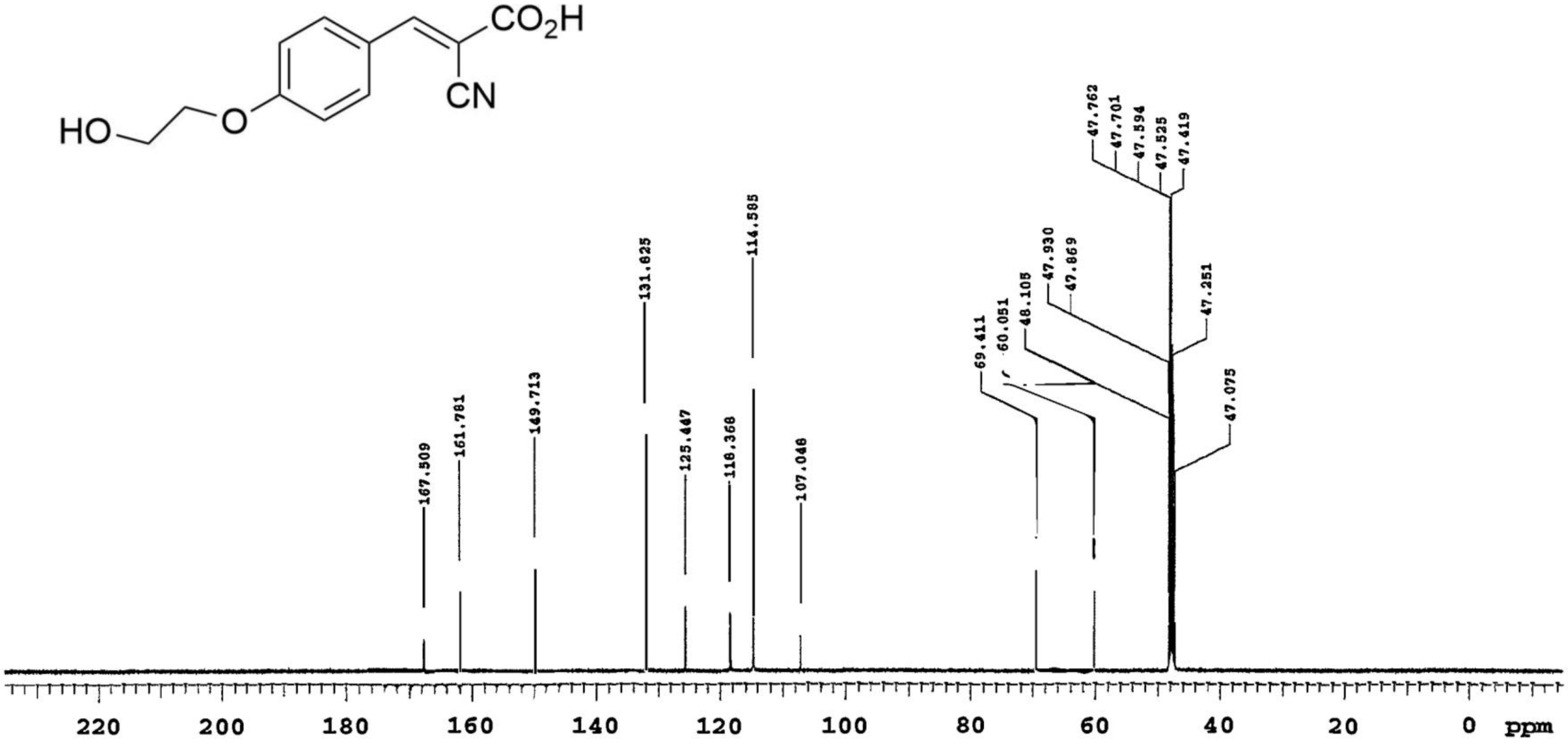

HO
O
CO2H
CN
167.509
161.781
149.713
131.625
125.447
118.368
114.585
107.046
69.411
60.051
48.105
47.930
47.869
47.762
47.701
47.594
47.525
47.419
47.251
47.075
220
200
180
160
140
120
100
80
60
40
20
0
ppm

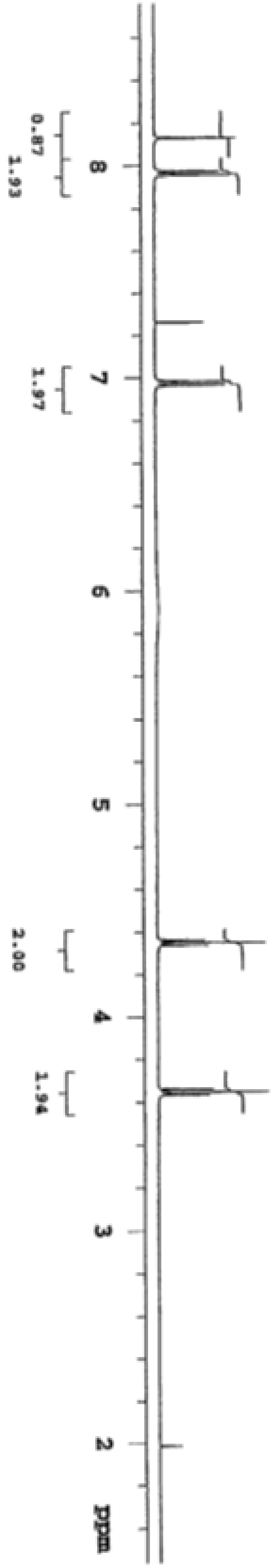
0.87
1.93
1.97
2.00
1.94
8
7
6
5
4
3
2
ppm

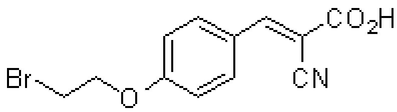
Br
O
CO2H
CN

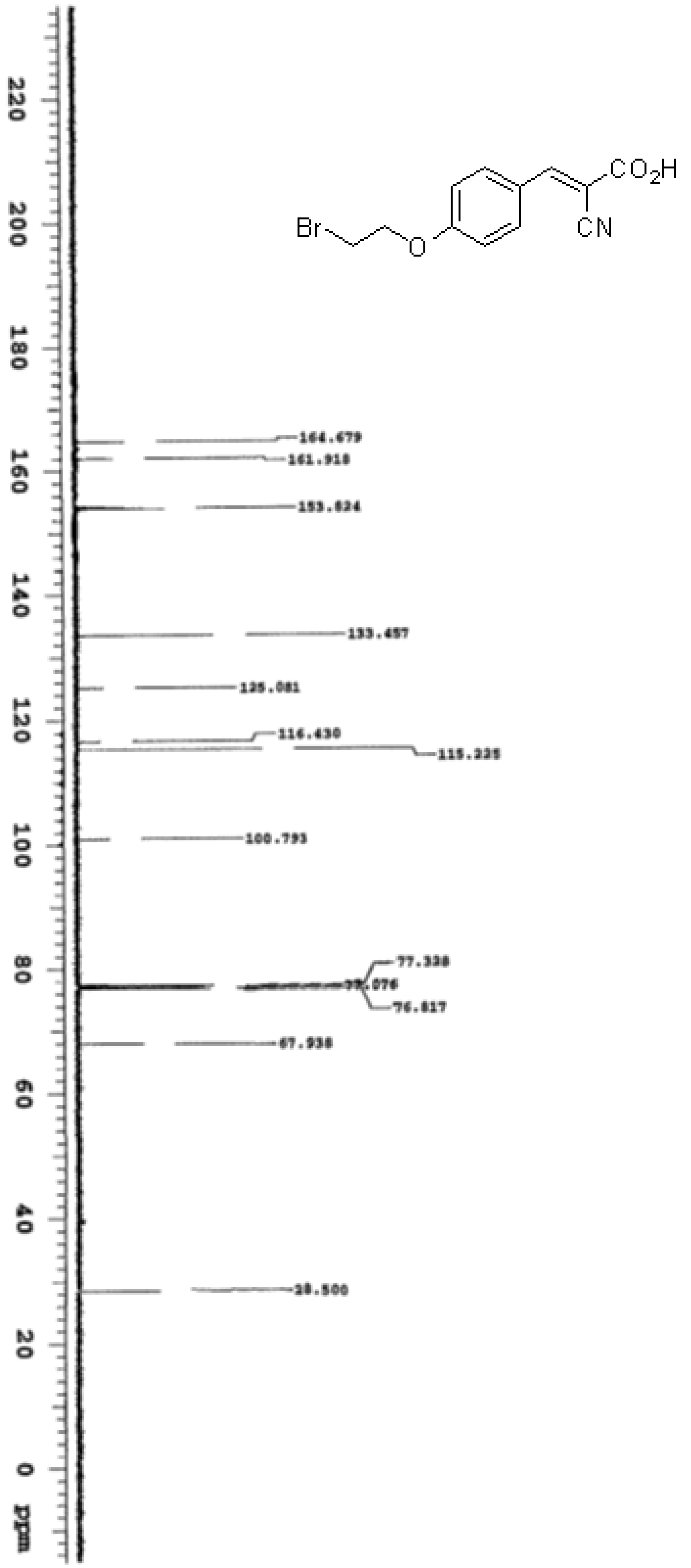

Br
O
CO2H
CN
164.679
161.918
133.457
116.430
100.793
220
200
180
160
140
120
100
80
60
40
20
0
ppm
